# Mechanism of Fei-Xian Formula in the Treatment of Pulmonary Fibrosis on the Basis of Network Pharmacology Analysis Combined with Molecular Docking Validation

**DOI:** 10.1155/2021/6658395

**Published:** 2021-08-03

**Authors:** Xiao-Li Chen, Cheng Tang, Qing-Ling Xiao, Zhong-Hua Pang, Dan-Dan Zhou, Jin Xu, Qi Wang, Ya-xi Zhao, Qi-Yong Zhu

**Affiliations:** ^1^Department of Respiratory Medicine, Affiliated Hospital of Integrated Traditional Chinese and Western Medicine, Nanjing University of Chinese Medicine, Nanjing 210028, Jiangsu Province, China; ^2^Laboratory of Cellular and Molecular Biology, Jiangsu Province Academy of Traditional Chinese Medicine, Nanjing 210028, Jiangsu Province, China; ^3^Department of Critical Care Medicine, Affiliated Hospital of Integrated Traditional Chinese and Western Medicine, Nanjing University of Chinese Medicine, Nanjing 210028, Jiangsu Province, China

## Abstract

**Objective:**

This study aimed to clarify the mechanism of Fei-Xian formula (FXF) in the treatment of pulmonary fibrosis based on network pharmacology analysis combined with molecular docking validation.

**Methods:**

Firstly, ingredients in FXF with pharmacological activities, together with specific targets, were identified based on the BATMA-TCM and TCMSP databases. Then, targets associated with pulmonary fibrosis, which included pathogenic targets as well as those known therapeutic targets, were screened against the CTD, TTD, GeneCards, and DisGeNet databases. Later, Cytoscape was employed to construct a candidate component-target network of FXF for treating pulmonary fibrosis. In addition, for nodes within the as-constructed network, topological parameters were calculated using CytoHubba plug-in, and the degree value (twice as high as the median degree value for all the nodes) was adopted to select core components as well as core targets of FXF for treating pulmonary fibrosis, which were subsequently utilized for constructing the core network. Furthermore, molecular docking study was carried out on those core active ingredients together with the core targets using AutoDock Vina for verifying results of network pharmacology analysis. At last, OmicShare was employed for enrichment analysis of the core targets.

**Results:**

Altogether 12 active ingredients along with 13 core targets were identified from our constructed core component-target network of FXF for the treatment of pulmonary fibrosis. As revealed by enrichment analysis, the 13 core targets mostly concentrated in regulating biological functions, like response to external stimulus (from oxidative stress, radiation, UV, chemical substances, and virus infection), apoptosis, cell cycle, aging, immune process, and protein metabolism. In addition, several pathways, like IL-17, AGE-RAGE, TNF, HIF-1, PI3K-AKT, NOD-like receptor, T/B cell receptor, and virus infection-related pathways, exerted vital parts in FXF in the treatment of pulmonary fibrosis.

**Conclusions:**

FXF can treat pulmonary fibrosis through a “multicomponent, multitarget, and multipathway” mean. Findings in this work lay foundation for further exploration of the FXF mechanism in the treatment of pulmonary fibrosis.

## 1. Introduction

Pulmonary fibrosis may take place in a variety of clinical settings and may threaten human life [1, 2]. It is featured by the disturbed cell homeostasis and changed cell composition in peripheral lungs, thus resulting in excess extracellular matrix (ECM) accumulation and lung dysfunction [3]. Over the last 10 years, investigators have identified that some molecular and cellular signal transduction pathways are related to the pulmonary fibrosis pathogenesis, which promotes to identify more novel therapeutic targets [4, 5]. Numerous treatments have been utilized to treat pulmonary fibrosis, but few of them elevate patient survival or improve their quality of life [6]. Nintedanib and pirfenidone have been approved to be used to treat pulmonary fibrosis, yet they have serious diverse reactions (such as gastrointestinal symptoms, photosensitivity, and abnormal liver function) [7–10]. Such phenomenon may be ascribed to the complicated regulatory networks involved in pulmonary fibrosis, which suppress or promote associated target genes or pathway expression [11–13]. It is increasingly suggested that epigenetic, genetic, or proteomic factors play important roles in these regulatory networks in pulmonary fibrosis [14–16]. Besides, the networks modulate fibrosis-related gene expression through activating or inactivating the relevant pathways, but not through one single pathway [17].

Traditional Chinese medicine (TCM) exerts multi-target effects and has been used to treat pulmonary fibrosis by regulating oxidant stress, inflammation, and so on. The TCM pathogenesis of pulmonary fibrosis is Yin deficiency and blood stasis. Thus, the treatment principle is dominated by promoting blood circulation to remove blood stasis, enriching Yin and nourishing the lung [18]. Fei-Xian formula (FXF), the cipher prescription used to treat pulmonary fibrosis at our institution, is made up of 7 herbs, Salviae Miltiorrhizae Radix et Rhizoma (Danshen, DS), Persicae Semen (Taoren, TR), Hirudo (Shuizhi, SZ), Paeoniae Radix Rubra (Chishao, CS), Asparagi Radix (Tiandong, TD), Canarii Fructus (Qingguo, QG), and Oroxyli Semen (Muhudie, MHD). FXF is effective in relieving the pulmonary fibrosis symptoms, improving lung function (evaluated by vital capacity max (VC Max), total lung capacity (TLC), diffusion capacity for carbon monoxide-single breath method (DLCO-SB), and diffusion capacity for carbon monoxide/alveolar volume (DLCO/VA)), and enhancing the exercise tolerance as well as quality of life of patients [19, 20]. Nonetheless, the multi-gene and multi-pathway mechanism of FXF in the treatment of pulmonary fibrosis remains largely unclear.

In the treatment of disease, the traditional Chinese medicine (TCM) herbs are less toxic. Nonetheless, the mechanism of TCM remains unknown so far, which may thus spark criticism as it becomes increasingly popular nowadays. Therefore, it is of great importance to investigate those target genes and pathways for the sake of modernizing TCM. Network pharmacology, a critical technical approach for investigating TCM formulas in systems biology, can visualize the herb and formula pharmacological effects and the underlying molecular mechanisms through multidisciplinary integration, including computer technology (CT), high-throughput omics, network database retrieval, and pharmacology [21, 22]. In the present work, network pharmacology analysis was adopted to predict pharmacodynamic material basis together with the underlying molecular mechanism of FXF for the treatment of pulmonary fibrosis, and the results were then validated by molecular docking study. Findings in this work lay theoretical basis to conduct experimental study and to apply FXF in clinic.

## 2. Materials and Methods

### 2.1. Mining the Potential Pharmacodynamic Compounds and Related Targets of FXF

The names of 7 herbs were imported into the BATMAN-TCM, Traditional Chinese Medicine Systems Pharmacology (TCMSP) database, and the analysis platform successively, to acquire the chemical compounds with corresponding information [23]. Thereafter, the drug likeness (DL) ≥ 0.18 and bioavailability (OB) ≥ 30% were used as the thresholds to screen those possible pharmacodynamic compounds in FXF by the method in literature [24, 25]. Additionally, certain compounds that did not conform to the selection thresholds but had great levels in each herb or had wide pharmacological actions, or were utilized to identify single medicine in the pharmacopeia, were incorporated as the possible pharmacodynamic compounds as well, including hirudin, salvianolic acid A, amygdalin, and oroxin [26–29]. Moreover, for those active components, the possible targets were discovered against the TCSMP database based on drug structural similarity assessment as well as reverse molecular docking, which were later used for constructing a potential FXF pharmacodynamic target set.

### 2.2. Pulmonary Fibrosis-Associated Target Screening

Targets associated with the pathogenesis or treatment of pulmonary fibrosis were acquired based on DisGeNet (https://www.disgenet.org/) [30], TTD (http://db.idrblab.net/ttd/) [31], CTD (http://ctdbase.org/) [32], and GeneCards (https://www.genecards.org/) [33] databases with the following keyword: “pulmonary fibrosis.” Targets were sorted out according to disease specificity index (DSI) from high to low against DisGeNet database, while targets larger than median were chosen. The abnormal drugs with related targets in TTD database were ruled out. For CTD database, those 200 most significant genes with the highest inference score were chosen. With regard to GeneCards, targets of Score ≥10 were chosen. Afterwards, targets retrieved based on these 4 databases were combined for the construction of a target set related to pulmonary fibrosis.

### 2.3. Establishment of the Core Component-Target Network of FXF for the Treatment of Pulmonary Fibrosis

Firstly, we chose the Uniprot database [34] in this work and chose species as “*Homo sapiens*”; then, target names acquired from these two steps were normalized for the acquisition of distinct Uniprot IDs and gene names. Later, both target sets were incorporated into Venny tool (https://bioinfogp.cnb.csic.es/tools/venny/), respectively, for obtaining overlapping targets, and they were regarded as the candidate FXF pharmacodynamic targets for the treatment of pulmonary fibrosis. Moreover, we applied Cytoscape in constructing the FXF candidate component-target network for the treatment of pulmonary fibrosis, where “component” was set as square, whereas “target” was set as circle. Thereafter, the node degree in candidate component-target network was calculated and ranked by CytoHubba plug-in [35], and the value (twice as high as the median degree value for the nodes) was adopted to select core components as well as core targets of FXF for treating pulmonary fibrosis, which were subsequently utilized for constructing the core network.

### 2.4. Compound-Target Interaction Validation

AutoDock Vina [36] (version: 1.1.2) was used to validate the relationships of compounds with targets. The compound mol2 structures were obtained based on TCMSP database, and target protein 3D structures were acquired from RCSB PDB database (http://www.rcsb.org/) [37]. Prior to molecular docking, proteins and ligands were prepared using AutoDockTools [38] (version: 1.5.6) and the MOE method. With regard to target proteins, their crystal structures were pre-treated, including removal of water molecules, 3D hydrogenation, protonation, correction of protein structure, energy optimization, and retention of target active region. Additionally, the ligand structures must conform to the low-energy conformation. Moreover, the box size and coordinates in molecular docking were finally determined based on ligand position. To achieve higher calculation accuracy, the exhaustiveness parameter was set at 20. The remaining parameters were the defaults except as otherwise noted. Thereafter, the components were integrated to the target proteins in a semiflexible manner, which produced altogether 9 conformations. The most affinal conformation was chosen to be the eventual docking conformation.

### 2.5. GO and KEGG Enrichment Analysis for Core Targets

For better exploring those biological processes (BPs), molecular functions (MFs), cellular components (CCs), and regulatory signal transduction pathways related to the FXF core targets for the treatment of pulmonary fibrosis, we input the information of selected core targets. Thereafter, GO-BP along with KEGG enrichment analysis was carried out on these targets using OmicShare online software [39] (https://www.omicshare.com/) at a *p* < 0.05 threshold.

## 3. Results

### 3.1. Selection of Potential Pharmacodynamic Components and Targets of FXF

By searching against the TCMSP database, altogether 201 chemical components were obtained from 7 herbs in FXF and 126 were selected as the core components upon the DL ≥ 0.18 and OB ≥ 30% thresholds. Additionally, compounds with lower OB or DL value that had great contents in each herb were also collected as the potential pharmacodynamic components of FXF. At last, DS, TR, SZ, CS, TD, QG, and MHD had 61, 23, 1, 27, 9, 6, and 19 potential pharmacodynamic components, separately, of which, baicalin, beta-sitosterol, ellagic acid, stigmasterol, quercetin, and 2,5-dihydroxy-6,7-dimethoxyflavone were widely distributed in several herbs.  Table S1 presents the information for the possible pharmacodynamic components of FXF.

Thereafter, the TCMSP database was searched to identify targets for the 130 potential pharmacodynamic components, and finally 286 were found (Table S2). DS, TR, SZ, CS, TD, QG, and MHD possessed 140, 71, 2, 114, 203, 207, and 131 potential targets, respectively. The 7 herbs obviously overlapped, regardless of the different target numbers in each herb, suggesting that those diverse herbs of FXF possibly exerted different actions by modulating similar targets.

**Figure 1:**
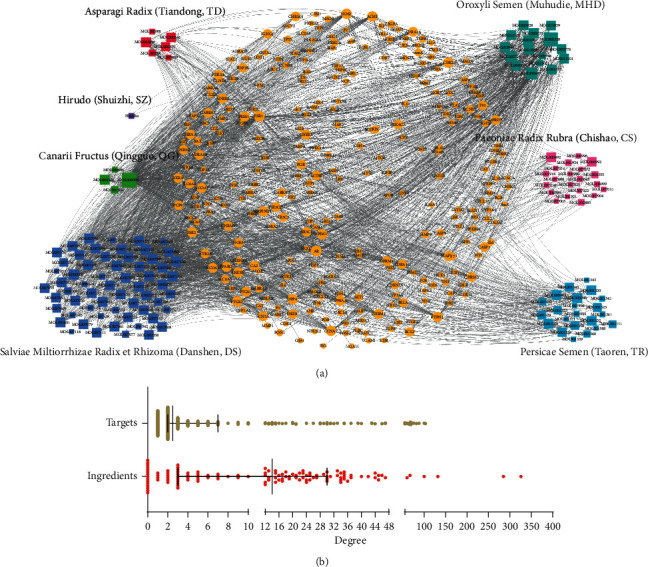
Establishment of the pharmacodynamic component-target network of FXF. (a) All components (compound ID) obtained from different herbs were associated with related targets for the construction of the compound-target network, in which one node represented one compound (different square colors indicated different herbs) and corresponding target (yellow circles). (b) The degree value distribution of nodes (ingredients and targets) in the network.

To systemically and holistically understand the component-target network of FXF, Cytoscape was utilized to establish a network map, including 2969 edges and 416 nodes (Figure 1(a)). The node degree in the map represented the target or edge number related to nodes based on topological analysis (Figure 1(b)). There were 65 components discovered in the as-constructed network upon the threshold of median ≥14 degrees. Quercetin, beta-sitosterol, stigmasterol, baicalein, and luteolin of these components functioned in 326, 285, 132, 100, and 67 targets, separately, and they might serve as the obvious core components of FXF.

### 3.2. Selection of Core Targets in FXF for the Treatment of Pulmonary Fibrosis

**Figure 2:**
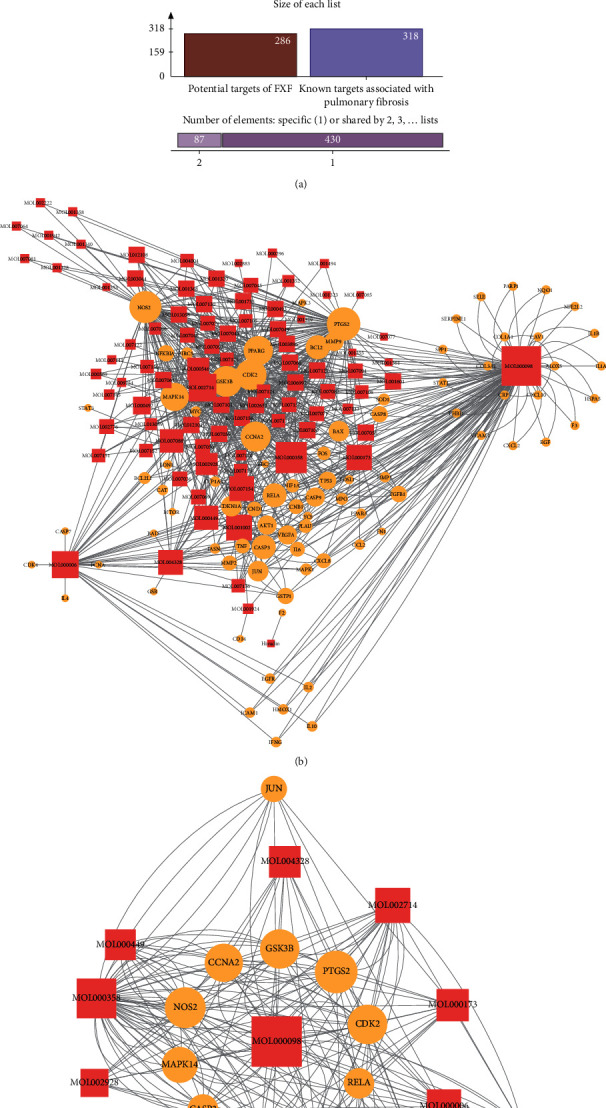
Screening of core targets for FXF in the treatment of pulmonary fibrosis. (a) As observed from the Venn diagram, there were 87 overlapping candidate targets between FXF and the known targets associated with pathological course in pulmonary fibrosis. (b) The candidate component-target network for FXF in the treatment of pulmonary fibrosis. (c) The core component-target network for FXF in the treatment of pulmonary fibrosis.

Pulmonary fibrosis represents one of the polygenic genetic diseases, and its pathogenesis may be studied by examining the interactions between genes or between genes and the environment. In this study, the databases including CTD, TTD, DisGeNet, and GeneCards were retrieved, which discovered 318 targets related to pulmonary fibrosis (Table S3). Besides, 87 of these discovered targets were also recognized as the potential targets for the pharmacodynamic components in FXF, which suggested that FXF had some therapeutic effect (Figure 2(a) and Table S4). These 87 targets may be the candidate targets of FXF in treating pulmonary fibrosis, while the 88 pharmacodynamic active components that exert regulatory effect on these targets are the candidate components.

Additionally, the candidate component-target network of FXF for the treatment of pulmonary fibrosis was constructed using Cytoscape (Figure 2(b)). For better selecting the FXF core components and core targets for the treatment of pulmonary fibrosis, we adopted the CytoHubba plug-in for calculating and sorting those node topological parameters (degrees) in the as-constructed network (Table S5). Thereafter, nodes with degree value greater than or equal to twice as high as the median degree value (=5) for all the nodes were selected as the core components and core targets in FXF for the treatment of pulmonary fibrosis. Later, the core component-core target network (including 12 core components and 13 core targets) was established (Figure 2(c)). Of these core components, quercetin, beta-sitosterol, baicalein, luteolin, ellagic acid, wogonin, naringenin, tanshinone IIa, stigmasterol, and cryptotanshinone from DS, TR, CS, TD, QG, and MHD in FXF were the most significant in line with the degree values. Some of them were suggested previously to postpone pulmonary fibrosis course and improve the symptoms [40–43]. Besides, PTGS2, NOS2, CDK2, GSK3B, CCNA2, PPARG, MAPK14, CASP3, BCL2, and RELA ranked the top 10 places based on the degree value.

### 3.3. Compound-Target Network Validation

**Figure 3:**
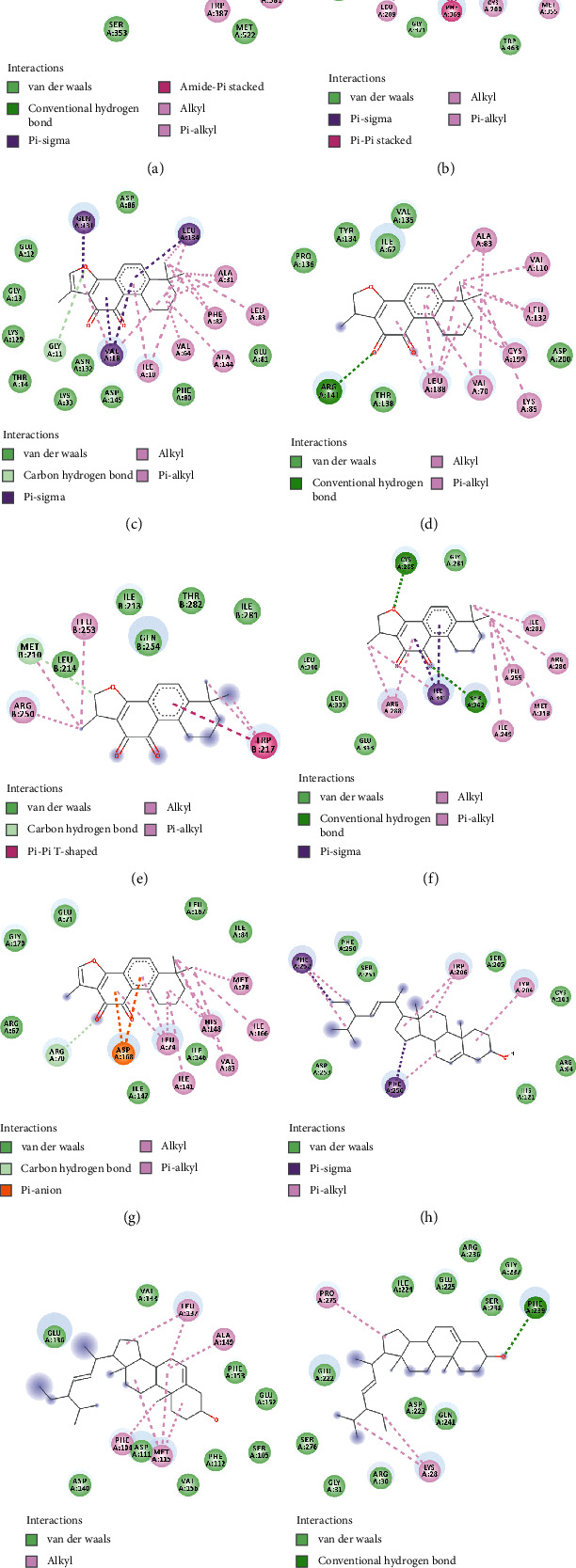
Virtual docking for core components and core targets in FXF for the treatment of pulmonary fibrosis. Virtual docking of cryptotanshinone with COX-2 (a), GSK-3 beta (d), Cyclin-A2 (e), and PPAR-gamma (f). Virtual docking of tanshinone IIa with iNOS (b), CDK2 (c), and MAPK14 (g). Virtual docking of stigmasterol with Caspase-3 (h), Bcl-2 (i), and p65 (j).

The relationships between components and targets were evaluated by molecular docking analysis, which helped to reduce network complexity while improving its accuracy. Thus, 10 core targets as well as 10 core compounds were discovered by molecular docking (Table 1 and Figure 3).

Thereafter, PTGS2, NOS2, CDK2, GSK3B, CCNA2, PPARG, MAPK14, CASP3, BCL2, and RELA were searched in the PDB protein database, respectively, to acquire 3D structures. It was observed from the results of binding free energy in Table 1 that most of the core compounds could tightly combine with core targets. Typically, stigmasterol is most closely bounded to BCL2 (Figure 3(i)), CASP3 (Figure 3(h)), and RELA (Figure 3(j)). In addition, cryptotanshinone was the compound with the most tight correlation with CCNA2 (Figure 3(e)), GSK3B (Figure 3(d)), PPARG (Figure 3(f)), and PTGS2 (Figure 3(a)). Meanwhile, tanshinone IIa was the compound with the lowest binding energy to CDK2 (Figure 3(c)), MAPK14 (Figure 3(g)), and NOS2 (Figure 3(b)) in this molecular docking experiment. In virtual docking (Figure 3(a) and Figure 3(d)-3(f)), cryptotanshinone formed the hydrogen bonds with ARG_120_, ARG_141_, MET_210_, and SER_342_ of COX-2, GSK-3 beta, Cyclin-A2, and PPAR-gamma, respectively. Meanwhile, tanshinone IIa formed the potent hydrophobic interaction with iNOS (*π*-sigma bonds with TRP_194_, Figure 3(b)), CDK2 (alkyl bonds with ALA_31_, Figure 3(c)), and MAP kinase 14 (alkyl bonds with MET_78_, Figure 3(g)). Typically, the stigmasterol-caspase-3 complex became stable via forming the *π*-sigma and *π*-alkyl with the PHE_252_ and PHE_256_ (Figure 3(h)). Correspondingly, the stigmasterol–Bcl-2 complex and stigmasterol-p65 complex got steady severally via forming hydrophobic interaction with MET_115_ residues (Figure 3(i)) and the hydrogen bond with PHE_239_ residues (Figure 3(j)).

### 3.4. Enrichment Analysis of FXF Core Targets for the Treatment of Pulmonary Fibrosis

**Figure 4:**
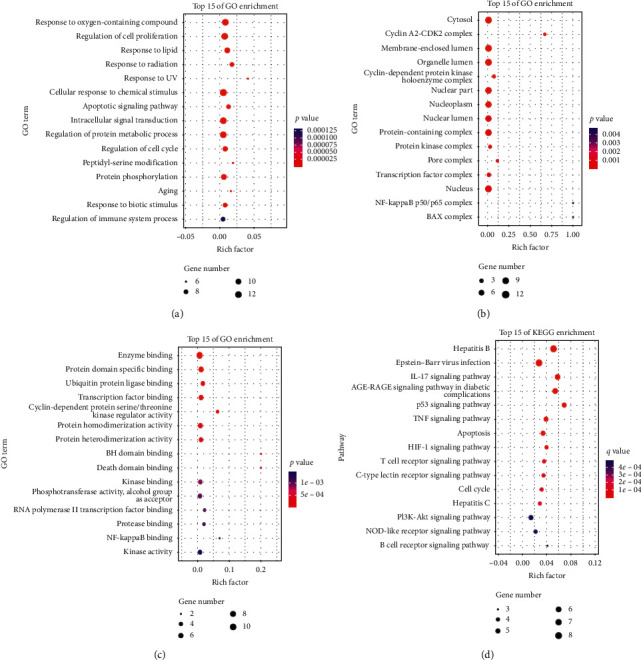
Enrichment analysis of core targets for FXF on the treatment of pulmonary fibrosis through OmicShare: (a) The top fifteen enriched GO-biological process; (b) GO-molecular functions; (c) GO-cellular components terms; and (d) KEGG pathways. The abscissa shows the enrichment factor, and the ordinate shows the GO terms or KEGG pathways. The color of the dot represents the adjusted *p*-value/*q*-value, and the size of the dot represents the number of core targets mapped to the reference GO terms or pathways.

For better understanding the multi-target and multi-pathway mechanism by which FXF treated pulmonary fibrosis, the OmicShare online approach was used to carry out GO as well as KEGG analysis on the thirteen core targets, to identify the biological processes/molecular functions along with signal transduction pathways in FXF for the treatment of pulmonary fibrosis (*p* < 0.05, FDR < 0.05). As shown, external stimulus (from oxidative stress, radiation, UV, chemical substances, and virus infection), apoptosis, cell cycle, aging, immune process, and protein metabolism were the mostly concentrated GO-BP terms (Figures 4(a)–4(c)). These core targets were mostly concentrated on several KEGG pathways, like IL-17, AGE-RAGE, TNF, HIF-1, PI3K-AKT, NOD-like receptor, T/B cell receptor, and virus infection-related pathways, indicating their important parts in the treatment of pulmonary fibrosis.

## 4. Discussion

Pulmonary fibrosis, an interstitial lung disease featured by the fibrotic, chronic, and progressive nature, is an uncommon disorder generally seen among the old people [44]. Its incidence in the North American and European countries is reported to be 2.8–9.3/100,000 persons. At present, few epidemiological data are available in China, yet the incidence of pulmonary fibrosis shows an increasing trend recently [45]. Usual interstitial pneumonia (UIP) is characteristic of pulmonary fibrosis on high-resolution chest CT or in patients with a pulmonary fibrosis history. As for the major clinical manifestations, pulmonary fibrosis mainly manifests as progressive dyspnea, hypoxia, restricted ventilation impairment, gas exchange disturbance, and respiratory failure [46]. For the time being, there is no radical treatment for pulmonary fibrosis, and treatment is mainly conducted for delaying disease progression, improving patient quality of life, and prolonging patient survival. Pirfenidone and nintedanib are the anti-fibrotic drugs adopted to treat pulmonary fibrosis, which are effective to some extent; however, their high prices and adverse reactions have restricted their application. Recently, Chinese herbs have exerted vital parts in treating pulmonary fibrosis. It is reported in numerous articles that Chinese herbs alleviate clinical symptoms, improve patient quality of life, and boost the exercise tolerance while delaying lung function decline among pulmonary fibrosis patients [47, 48].

Chinese herbs represent an important TCM means to treat diseases, which has been utilized due to the complicated pulmonary fibrosis pathogenesis as well as the common patient syndrome [49]. Pulmonary fibrosis can be classified as Yin deficiency and internal heat syndrome, lung and kidney qi deficiency syndrome, phlegm turbid/heat obstruction of lung syndrome, and blood stasis syndrome, based on the diffuse interstitial lung disease diagnostic criteria in TCM (2012 Edition). Typically, clinical medication mainly aims to promote qi, accelerate blood circulation, eliminate blood stasis, and reinforce Yin. Therefore, FXF, which has the effect of nourishing Yin and removing blood stasis, is used as a cipher prescription in our hospital for the treatment of pulmonary fibrosis.

Herbal medicine has different and complicated components. Previous research on herbal medicine is still encountered with certain problems, like the complicated composition and the unknown mechanism. Network pharmacological analysis has been extensively adopted to investigate the pharmacological mechanisms of TCM currently. As a result, the holistic view in TCM was used in combination with network pharmacology analysis and the syndrome differentiation science system in this study to identify core pharmacodynamic material basis and targets. Then, molecular docking study was carried out on those core active ingredients together with the core targets using AutoDock Vina for verifying results of network pharmacology analysis. Finally, these core targets were subjected to GO and pathway enrichment analysis.

As revealed in this work, quercetin, beta-sitosterol, baicalein, luteolin, ellagic acid, wogonin, naringenin, tanshinone IIa, stigmasterol, and cryptotanshinone were identified as the core compounds for treating pulmonary fibrosis. Some studies demonstrate the effect of quercetin, baicalein, tanshinone IIA, and cryptotanshinone on suppressing experimental pulmonary fibrosis induced by bleomycin or silica through promoting lung tissue self-healing, modulating disturbed redox-balance in lung tissue, alleviating local inflammation, reducing collagen deposition, promoting ECM degradation, inducing pulmonary fibrosis cell apoptosis, reversing angiotensin production, and regulating multiple signaling molecules and pathways (such as TGF-beta, sphingosine kinase, SMAD, Nrf2, STAT3) [41, 42, 50–54]. Our research results were consistent with those in previous study, indicating that these compounds were the core effective components of FXF to treat pulmonary fibrosis.

According to the node degrees in our constructed core component-target network, PTGS2, NOS2, CDK2, GSK3B, CCNA2, PPARG, MAPK14, CASP3, BCL2, and RELA were suggested as core targets of FXF for the treatment of pulmonary fibrosis. In addition, molecular docking was conducted to validate the associations of core components with core targets. Upon GO-BP annotation analysis, the core targets exerted vital parts in external stimulus (from oxidative stress, radiation, UV, chemical substances, and virus infection), apoptosis, cell cycle, aging, immune process, and protein metabolism. As revealed by KEGG pathway analysis, numerous pathways were tightly associated with the pulmonary fibrosis pathogenic mechanism. Typically, IL-17, AGE-RAGE, TNF, HIF-1, PI3K-AKT, NOD-like receptor, T/B cell receptor, and virus (EBV and hepatitis virus) infection-related pathways were the most significant pathways involved. The associations of diverse pathways, including EBV and hepatitis virus infections, with pulmonary fibrosis are identified through fundamental clinical studies to induce pulmonary fibrosis [55–57]. Meanwhile, epithelial-mesenchymal transition (EMT) in pulmonary fibrosis may also be related to activating the NOD-like receptor protein (NLRP) 1 and NLRP3 inflammatory pathways [58]. The advanced glycation end products (AGE) may be obtained from non-enzymatic reaction of proteins and lipids with various oxidants in the process of aging. In addition, the receptor for AGE (RAGE) has been demonstrated to be associated with alveolar homeostasis as well as pulmonary fibrosis. The aberrant epithelial stromal repair capacity in pulmonary fibrosis is linked with this aging process. The elevated AGE-RAGE proportion in pulmonary fibrosis is correlated with epithelial stromal repair during pulmonary fibrosis [59, 60]. In the pathological process of pulmonary fibrosis, the infiltration of various immune cells and the release of immuno-inflammatory factors play an important role, which is also considered as a new therapeutic target to reverse the disease, such as IL-17 [61] and T cell receptor [62]. In addition, the mitochondria-mediated apoptosis also exerts a vital part during pulmonary fibrosis genesis and progression mediated by both mitochondria and ER stress (triggered by HIF-1alpha) [63–65].

Nonetheless, certain limitations should be noted in the present work [66], like the data-based network pharmacology, which made it impossible to examine whether all data were comprehensively collected and whether the criteria to select core components were completely accurate. Besides, the efficacy of additional components and effects of dosage should be further examined.

## 5. Conclusion

To sum up, the mechanism of action by which FXF treats pulmonary fibrosis may be related to the regulation of several pathways, like IL-17, AGE-RAGE, TNF, HIF-1, PI3K-AKT, NOD-like receptor, T/B cell receptor, and virus infection-related pathways.

## Figures and Tables

**Table 1:** Virtual docking of core components with core targets in FXF for the treatment of pulmonary fibrosis.

Core ingredients	Binding energy (kcal·mol^−1^)									
	BCL2	CASP3	CCNA2	CDK2	GSK3B	MAPK14	NOS2	PPARG	PTGS2	RELA
MOL000006	−7.2	−6.8	−6.8	−10	−8	−8.9	−8.9	−7.2	−7.1	−6.2
MOL000098	−7.2	−6.5	−6.8	−10.1	−8	−8	−8.7	−7.2	−6.6	−6.1
MOL000173	−7.3	−6.6	−6.1	−10	−7.8	−7.8	−9.9	−7.3	−6.9	−6
MOL000358	−7.6	−7.5	−6.8	−7.7	−9.1	−9.3	−9.4	−8.7	−4.5	−6.7
MOL000449	−**8**	−**8.1**	−6.6	−5	−8.9	−8.4	−9.1	−7.7	−3.2	−**7.1**
MOL001002	−6.3	−6.3	−6	−10.4	−8.4	−8.1	−8.7	−7.3	−6.8	−6.3
MOL002714	−7.1	−6.9	−6.5	−10.1	−7.9	−8.2	−9.9	−7.1	−6.9	−6.2
MOL004328	−7	−7	−6	−6.7	−7.1	−7.7	−7.7	−7.4	−5.8	−6.5
MOL007088	−7.7	−7.8	−**7**	−10.8	−**9.5**	−9.3	−10.5	−**8.8**	−**9.2**	−6.9
MOL007154	−7.7	−7.7	−6.8	−**10.9**	−9.3	−**9.4**	−**10.6**	−8.4	−8.8	−6.7

## Data Availability

The datasets used and/or analyzed during the current study are available from the corresponding author on reasonable request.

## Supplementary Materials

Supplementary Materials Table S1: all the pharmacodynamic ingredients of FXF. Table S2: all the potential pharmacodynamic targets of FXF. Table S3: known pulmonary fibrosis-related targets. Table S4: FXF shared 87 potential pharmacodynamic targets with known pulmonary fibrosis-related targets. Table S5: degree values of nodes in the candidate active ingredient-target network of FXF in treating pulmonary fibrosis.
