# Supplementary material for: Mechanism of Fei-Xian Formula in the Treatment of Pulmonary Fibrosis on the Basis of Network Pharmacology Analysis Combined with Molecular Docking Validation
Source: Evid Based Complement Alternat Med. 2021 Aug 3;2021:6658395. doi: 10.1155/2021/6658395 (PMC8357467; doi:10.1155/2021/6658395)
Supplement: Supplementary Materials — Table S1: all the pharmacodynamic ingredients of FXF. Table S2: all the potential pharmacodynamic targets of FXF. Table S3: known pulmonary fibrosis-related targets. Table S4: FXF shared 87 potential pharmacodynamic targets with known pulmonary fibrosis-related targets. Table S5: degree values of nodes in the candidate active ingredient-target network of FXF in treating pulmonary fibrosis. [file 6658395.f1.zip › 6658395.f1/Table S1.docx]

| **Table S1. All the pharmacodynamic ingredients of FXF** | | | | |
| --- | --- | --- | --- | --- |
| **Herb Name** | **Molecule ID** | **Molecule Name** | **OB (%)** | **DL** |
| Salviae Miltiorrhizae Radix et Rhizoma (Danshen, DS) | MOL001601 | 1,2,5,6-tetrahydrotanshinone | 38.75 | 0.36 |
|  | MOL001659 | Poriferasterol | 43.83 | 0.76 |
|  | MOL001771 | poriferast-5-en-3beta-ol | 36.91 | 0.75 |
|  | MOL001942 | isoimperatorin | 45.46 | 0.23 |
|  | MOL002222 | sugiol | 36.11 | 0.28 |
|  | MOL002651 | Dehydrotanshinone II A | 43.76 | 0.4 |
|  | MOL002776 | Baicalin | 40.12 | 0.75 |
|  | MOL000569 | digallate | 61.85 | 0.26 |
|  | MOL000006 | luteolin | 36.16 | 0.25 |
|  | MOL006824 | α-amyrin | 39.51 | 0.76 |
|  | MOL007036 | 5,6-dihydroxy-7-isopropyl-1,1-dimethyl-2,3-dihydrophenanthren-4-one | 33.77 | 0.29 |
|  | MOL007041 | 2-isopropyl-8-methylphenanthrene-3,4-dione | 40.86 | 0.23 |
|  | MOL007045 | 3α-hydroxytanshinoneⅡa | 44.93 | 0.44 |
|  | MOL007048 | (E)-3-[2-(3,4-dihydroxyphenyl)-7-hydroxy-benzofuran-4-yl]acrylic acid | 48.24 | 0.31 |
|  | MOL007049 | 4-methylenemiltirone | 34.35 | 0.23 |
|  | MOL007050 | 2-(4-hydroxy-3-methoxyphenyl)-5-(3-hydroxypropyl)-7-methoxy-3-benzofurancarboxaldehyde | 62.78 | 0.4 |
|  | MOL007051 | 6-o-syringyl-8-o-acetyl shanzhiside methyl ester | 46.69 | 0.71 |
|  | MOL007058 | formyltanshinone | 73.44 | 0.42 |
|  | MOL007059 | 3-beta-Hydroxymethyllenetanshiquinone | 32.16 | 0.41 |
|  | MOL007061 | Methylenetanshinquinone | 37.07 | 0.36 |
|  | MOL007063 | przewalskin a | 37.11 | 0.65 |
|  | MOL007064 | przewalskin b | 110.32 | 0.44 |
|  | MOL007068 | Przewaquinone B | 62.24 | 0.41 |
|  | MOL007069 | przewaquinone c | 55.74 | 0.4 |
|  | MOL007071 | przewaquinone f | 40.31 | 0.46 |
|  | MOL007077 | sclareol | 43.67 | 0.21 |
|  | MOL007079 | tanshinaldehyde | 52.47 | 0.45 |
|  | MOL007081 | Danshenol B | 57.95 | 0.56 |
|  | MOL007082 | Danshenol A | 56.97 | 0.52 |
|  | MOL007085 | Salvilenone | 30.38 | 0.38 |
|  | MOL007088 | cryptotanshinone | 52.34 | 0.4 |
|  | MOL007093 | dan-shexinkum d | 38.88 | 0.55 |
|  | MOL007094 | danshenspiroketallactone | 50.43 | 0.31 |
|  | MOL007098 | deoxyneocryptotanshinone | 49.4 | 0.29 |
|  | MOL007100 | dihydrotanshinlactone | 38.68 | 0.32 |
|  | MOL007101 | dihydrotanshinoneⅠ | 45.04 | 0.36 |
|  | MOL007105 | epidanshenspiroketallactone | 68.27 | 0.31 |
|  | MOL007108 | isocryptotanshi-none | 54.98 | 0.39 |
|  | MOL007111 | Isotanshinone II | 49.92 | 0.4 |
|  | MOL007115 | manool | 45.04 | 0.2 |
|  | MOL007118 | microstegiol | 39.61 | 0.28 |
|  | MOL007119 | miltionone Ⅰ | 49.68 | 0.32 |
|  | MOL007120 | miltionone Ⅱ | 71.03 | 0.44 |
|  | MOL007121 | miltipolone | 36.56 | 0.37 |
|  | MOL007122 | Miltirone | 38.76 | 0.25 |
|  | MOL007123 | miltirone Ⅱ | 44.95 | 0.24 |
|  | MOL007124 | neocryptotanshinone ii | 39.46 | 0.23 |
|  | MOL007125 | neocryptotanshinone | 52.49 | 0.32 |
|  | MOL007127 | 1-methyl-8,9-dihydro-7H-naphtho[5,6-g]benzofuran-6,10,11-trione | 34.72 | 0.37 |
|  | MOL007130 | prolithospermic acid | 64.37 | 0.31 |
|  | MOL007141 | salvianolic acid g | 45.56 | 0.61 |
|  | MOL007142 | salvianolic acid j | 43.38 | 0.72 |
|  | MOL007143 | salvilenone Ⅰ | 32.43 | 0.23 |
|  | MOL007145 | salviolone | 31.72 | 0.24 |
|  | MOL007150 | (6S)-6-hydroxy-1-methyl-6-methylol-8,9-dihydro-7H-naphtho[8,7-g]benzofuran-10,11-quinone | 75.39 | 0.46 |
|  | MOL007151 | Tanshindiol B | 42.67 | 0.45 |
|  | MOL007152 | Przewaquinone E | 42.85 | 0.45 |
|  | MOL007154 | tanshinone iia | 49.89 | 0.4 |
|  | MOL007155 | (6S)-6-(hydroxymethyl)-1,6-dimethyl-8,9-dihydro-7H-naphtho[8,7-g]benzofuran-10,11-dione | 65.26 | 0.45 |
|  | MOL007156 | tanshinone Ⅵ | 45.64 | 0.3 |
|  | MOL007136 | salvianolic acid a | 2.96 | 0.7 |
| Persicae Semen (Taoren, TR) | MOL001323 | Sitosterol alpha1 | 43.28 | 0.78 |
|  | MOL001328 | 2,3-didehydro GA70 | 63.29 | 0.5 |
|  | MOL001329 | 2,3-didehydro GA77 | 88.08 | 0.53 |
|  | MOL001339 | GA119 | 76.36 | 0.49 |
|  | MOL001340 | GA120 | 84.85 | 0.45 |
|  | MOL001342 | GA121-isolactone | 72.7 | 0.54 |
|  | MOL001343 | GA122 | 64.79 | 0.5 |
|  | MOL001344 | GA122-isolactone | 88.11 | 0.54 |
|  | MOL001348 | gibberellin 17 | 94.64 | 0.49 |
|  | MOL001350 | GA30 | 61.72 | 0.54 |
|  | MOL001351 | Gibberellin A44 | 101.61 | 0.54 |
|  | MOL001352 | GA54 | 64.21 | 0.53 |
|  | MOL001353 | GA60 | 93.17 | 0.53 |
|  | MOL001355 | GA63 | 65.54 | 0.54 |
|  | MOL001358 | gibberellin 7 | 73.8 | 0.5 |
|  | MOL001360 | GA77 | 87.89 | 0.53 |
|  | MOL001361 | GA87 | 68.85 | 0.57 |
|  | MOL001368 | 3-O-p-coumaroylquinic acid | 37.63 | 0.29 |
|  | MOL001371 | Populoside_qt | 108.89 | 0.2 |
|  | MOL000296 | hederagenin | 36.91 | 0.75 |
|  | MOL000358 | beta-sitosterol | 36.91 | 0.75 |
|  | MOL000493 | campesterol | 37.58 | 0.71 |
|  | MOL001320 | Amygdalin | 4.42 | 0.61 |
| Hirudo (Shuizhi, SZ) | MOLhirudin | hirudin | unknown | unknown |
| Paeoniae Radix Rubra (Chishao, CS) | MOL001002 | ellagic acid | 43.06 | 0.43 |
|  | MOL001918 | paeoniflorgenone | 87.59 | 0.37 |
|  | MOL001921 | Lactiflorin | 49.12 | 0.8 |
|  | MOL001924 | paeoniflorin | 53.87 | 0.79 |
|  | MOL002714 | baicalein | 33.52 | 0.21 |
|  | MOL002776 | Baicalin | 40.12 | 0.75 |
|  | MOL000358 | beta-sitosterol | 36.91 | 0.75 |
|  | MOL000359 | sitosterol | 36.91 | 0.75 |
|  | MOL004355 | Spinasterol | 42.98 | 0.76 |
|  | MOL000449 | Stigmasterol | 43.83 | 0.76 |
|  | MOL000492 | (+)-catechin | 54.83 | 0.24 |
|  | MOL006990 | (1S,2S,4R)-trans-2-hydroxy-1,8-cineole-B-D-glucopyranoside | 30.25 | 0.27 |
|  | MOL006992 | (2R,3R)-4-methoxyl-distylin | 59.98 | 0.3 |
|  | MOL006994 | 1-o-beta-d-glucopyranosyl-8-o-benzoylpaeonisuffrone_qt | 36.01 | 0.3 |
|  | MOL006996 | 1-o-beta-d-glucopyranosylpaeonisuffrone_qt | 65.08 | 0.35 |
|  | MOL006999 | stigmast-7-en-3-ol | 37.42 | 0.75 |
|  | MOL007003 | benzoyl paeoniflorin | 31.14 | 0.54 |
|  | MOL007004 | Albiflorin | 30.25 | 0.77 |
|  | MOL007008 | 4-ethyl-paeoniflorin_qt | 56.87 | 0.44 |
|  | MOL007012 | 4-o-methyl-paeoniflorin_qt | 56.7 | 0.43 |
|  | MOL007014 | 8-debenzoylpaeonidanin | 31.74 | 0.45 |
|  | MOL007016 | Paeoniflorigenone | 65.33 | 0.37 |
|  | MOL007018 | 9-ethyl-neo-paeoniaflorin A_qt | 64.42 | 0.3 |
|  | MOL007022 | evofolinB | 64.74 | 0.22 |
|  | MOL007025 | isobenzoylpaeoniflorin | 31.14 | 0.54 |
|  | MOL002883 | Ethyl oleate (NF) | 32.4 | 0.19 |
|  | MOL005043 | campest-5-en-3beta-ol | 37.58 | 0.71 |
| Asparagi Radix (Tiandong, TD) | MOL000358 | beta-sitosterol | 36.91 | 0.75 |
|  | MOL000359 | sitosterol | 36.91 | 0.75 |
|  | MOL003889 | methylprotodioscin_qt | 35.12 | 0.86 |
|  | MOL003891 | pseudoprotodioscin_qt | 37.93 | 0.87 |
|  | MOL003896 | 7-Methoxy-2-methyl isoflavone | 42.56 | 0.2 |
|  | MOL003901 | Asparaside A_qt | 30.6 | 0.86 |
|  | MOL000449 | Stigmasterol | 43.83 | 0.76 |
|  | MOL000546 | diosgenin | 80.88 | 0.81 |
|  | MOL000098 | quercetin | 46.43 | 0.28 |
| Canarii Fructus (Qingguo, QG) | MOL001002 | ellagic acid | 43.06 | 0.43 |
|  | MOL001494 | Mandenol | 42 | 0.19 |
|  | MOL000358 | beta-sitosterol | 36.91 | 0.75 |
|  | MOL004328 | naringenin | 59.29 | 0.21 |
|  | MOL006696 | α-amyrin acetate | 42.7 | 0.74 |
|  | MOL000098 | quercetin | 46.43 | 0.28 |
| Oroxyli Semen (Muhudie, MHD) | MOL001002 | ellagic acid | 43.06 | 0.43 |
|  | MOL012101 | Mosloflavone | 34.04 | 0.26 |
|  | MOL012108 | Negletein | 41.16 | 0.23 |
|  | MOL013058 | 2,5-dihydroxy-6,7-dimethoxyflavone | 41.52 | 0.28 |
|  | MOL013059 | 3,7,3',5'-tetramethoxy-2hydrochroxyflavone | 53.26 | 0.42 |
|  | MOL013061 | Baicalein-6-glucuronide | 38.72 | 0.76 |
|  | MOL000173 | wogonin | 30.68 | 0.23 |
|  | MOL001735 | Dinatin | 30.97 | 0.27 |
|  | MOL002714 | baicalein | 33.52 | 0.21 |
|  | MOL002776 | Baicalin | 40.12 | 0.75 |
|  | MOL002928 | oroxylin a | 41.37 | 0.23 |
|  | MOL003044 | Chryseriol | 35.85 | 0.27 |
|  | MOL000358 | beta-sitosterol | 36.91 | 0.75 |
|  | MOL004004 | 6-OH-Luteolin | 46.93 | 0.28 |
|  | MOL000449 | Stigmasterol | 43.83 | 0.76 |
|  | MOL013058 | 2,5-dihydroxy-6,7-dimethoxyflavone | 41.52 | 0.28 |
|  | MOL013059 | 3,7,3',5'-tetramethoxy-2hydrochroxyflavone | 53.26 | 0.42 |
|  | MOL013061 | Baicalein-6-glucuronide | 38.72 | 0.76 |
|  | MOL009754 | Oroxin | 9.58 | 0.72 |
