# Supplementary material for: Mechanism of Fei-Xian Formula in the Treatment of Pulmonary Fibrosis on the Basis of Network Pharmacology Analysis Combined with Molecular Docking Validation
Source: Evid Based Complement Alternat Med. 2021 Aug 3;2021:6658395. doi: 10.1155/2021/6658395 (PMC8357467; doi:10.1155/2021/6658395)
Supplement: Supplementary Materials — Table S1: all the pharmacodynamic ingredients of FXF. Table S2: all the potential pharmacodynamic targets of FXF. Table S3: known pulmonary fibrosis-related targets. Table S4: FXF shared 87 potential pharmacodynamic targets with known pulmonary fibrosis-related targets. Table S5: degree values of nodes in the candidate active ingredient-target network of FXF in treating pulmonary fibrosis. [file 6658395.f1.zip › 6658395.f1/Table S2.docx]

| **Table S2. All the potential pharmacodynamic targets of FXF** | | |
| --- | --- | --- |
| **Target ID** | **Annotation** | **Gene name** |
| TAR00003 | Nitric oxide synthase, inducible | NOS2 |
| TAR00006 | Prostaglandin G/H synthase 1 | PTGS1 |
| TAR00016 | Muscarinic acetylcholine receptor M3 | CHRM3 |
| TAR00017 | Thrombin | SERPIND1 |
| TAR00038 | Muscarinic acetylcholine receptor M1 | CHRM1 |
| TAR00046 | Estrogen receptor | ESR1 |
| TAR00048 | Androgen receptor | AR |
| TAR00070 | Sodium channel protein type 5 subunit alpha | SCN5A |
| TAR00078 | Peroxisome proliferator activated receptor gamma | PPARG |
| TAR00087 | Muscarinic acetylcholine receptor M5 | CHRM5 |
| TAR00094 | Prostaglandin G/H synthase 2 | PTGS2 |
| TAR00095 | Nitric-oxide synthase, endothelial | NOS3 |
| TAR00113 | 5-hydroxytryptamine receptor 3A | HTR3A |
| TAR00117 | Carbonic anhydrase II | CA2 |
| TAR00154 | Muscarinic acetylcholine receptor M4 | CHRM4 |
| TAR00158 | Retinoic acid receptor RXR-alpha | RXRA |
| TAR00163 | Delta-type opioid receptor | OPRD1 |
| TAR00165 | Acetylcholinesterase | ACHE |
| TAR00172 | CGMP-inhibited 3',5'-cyclic phosphodiesterase A | PDE3A |
| TAR00191 | Alpha-1A adrenergic receptor | ADRA1A |
| TAR00210 | Muscarinic acetylcholine receptor M2 | CHRM2 |
| TAR00216 | Alpha-1B adrenergic receptor | ADRA1B |
| TAR00229 | mRNA of Protein-tyrosine phosphatase, non-receptor type 1 | PTPN1 |
| TAR00239 | Sodium-dependent dopamine transporter | SLC6A3 |
| TAR00261 | Beta-2 adrenergic receptor | ADRB2 |
| TAR00272 | Alpha-1D adrenergic receptor | ADRA1D |
| TAR00287 | DNA topoisomerase II | TOP2B |
| TAR00299 | Mu-type opioid receptor | OPRM1 |
| TAR00307 | Estrogen receptor beta | ESR2 |
| TAR00309 | Gamma-aminobutyric acid receptor subunit alpha-1 | GABRA1 |
| TAR00332 | Dipeptidyl peptidase IV | DPP4 |
| TAR00402 | Mitogen-activated protein kinase 14 | MAPK14 |
| TAR00422 | Glycogen synthase kinase-3 beta | GSK3B |
| TAR00444 | Heat shock protein HSP 90 | HSP90AA1 |
| TAR00482 | Cell division protein kinase 2 | CDK2 |
| TAR00581 | Neuronal acetylcholine receptor protein, alpha-7 chain | CHRNA7 |
| TAR02928 | Trypsin-1 | PRSS1 |
| TAR02966 | Proto-oncogene serine/threonine-protein kinase Pim-1 | PIM1 |
| TAR03025 | Cyclin-A2 | CCNA2 |
| TAR03276 | Nuclear receptor coactivator 2 | NCOA2 |
| TAR03279 | Nuclear receptor coactivator 1 | NCOA1 |
| TAR00007 | Dopamine D1 receptor | DRD1 |
| TAR00175 | 5-hydroxytryptamine 2A receptor | HTR2A |
| TAR00290 | Sodium-dependent serotonin transporter | SLC6A4 |
| TAR00647 | Serine/threonine-protein kinase Chk1 | CHEK1 |
| TAR01201 | Ig gamma-1 chain C region | IGHG1 |
| TAR00209 | Progesterone receptor | PGR |
| TAR00252 | Mineralocorticoid receptor | NR3C2 |
| TAR00308 | Glucocorticoid receptor | NR3C1 |
| TAR00292 | D(2) dopamine receptor | DRD2 |
| TAR00079 | Coagulation factor Xa | F10 |
| TAR00288 | Aldose reductase | AKR1B1 |
| TAR00699 | mRNA of PKA Catalytic Subunit C-alpha | PRKACA |
| TAR00491 | Phosphatidylinositol-4,5-bisphosphate 3-kinase catalytic subunit, gamma isoform | PIK3CG |
| TAR04565 | Transcription factor p65 | RELA |
| TAR00298 | Epidermal growth factor receptor | EGFR |
| TAR04490 | RAC-alpha serine/threonine-protein kinase | AKT1 |
| TAR00740 | Vascular endothelial growth factor A | VEGFA |
| TAR04214 | G1/S-specific cyclin-D1 | CCND1 |
| TAR04054 | Bcl-2-like protein 1 | BCL2L1 |
| TAR04141 | Cyclin-dependent kinase inhibitor 1 | CDKN1A |
| TAR04090 | Caspase-9 | CASP9 |
| TAR00238 | 72 kDa type IV collagenase | MMP2 |
| TAR04334 | Matrix metalloproteinase-9 | MMP9 |
| TAR00354 | Mitogen-activated protein kinase 1 | MAPK1 |
| TAR04292 | Interleukin-10 | IL10 |
| TAR02915 | Retinoblastoma-associated protein | RB1 |
| TAR00573 | Cell division protein kinase 4 | CDK4 |
| TAR00265 | Tumor necrosis factor | TNF |
| TAR00414 | Transcription factor AP-1 | JUN |
| TAR00351 | Interleukin-6 | IL6 |
| TAR04087 | Caspase-3 | CASP3 |
| TAR00646 | Cellular tumor antigen p53 | TP53 |
| TAR04394 | NF-kappa-B inhibitor alpha | NFKBIA |
| TAR00568 | Xanthine dehydrogenase/oxidase | XDH |
| TAR01293 | DNA topoisomerase 1 | TOP1 |
| TAR04179 | E3 ubiquitin-protein ligase Mdm2 | MDM2 |
| TAR00648 | Amyloid beta A4 protein | APBA3 |
| TAR00353 | Interstitial collagenase | MMP1 |
| TAR04450 | Proliferating cell nuclear antigen | PCNA |
| TAR04496 | Receptor tyrosine-protein kinase erbB-2 | ERBB2 |
| TAR02132 | Heme oxygenase 1 | HMOX1 |
| TAR03575 | Caspase-7 | CASP7 |
| TAR04287 | Intercellular adhesion molecule 1 | ICAM1 |
| TAR04269 | Induced myeloid leukemia cell differentiation protein Mcl-1 | MCL1 |
| TAR04044 | Baculoviral IAP repeat-containing protein 5 | BIRC5 |
| TAR03978 | Interleukin-2 | IL2 |
| TAR04219 | G2/mitotic-specific cyclin-B1 | CCNB1 |
| TAR04590 | Tyrosinase | TYR |
| TAR00365 | Interferon gamma | IFNG |
| TAR04301 | Interleukin-4 | IL4 |
| TAR04172 | DNA topoisomerase 2-alpha | TOP2A |
| TAR00733 | Glutathione S-transferase P | GSTP1 |
| TAR04043 | Baculoviral IAP repeat-containing protein 4 | XIAP |
| TAR04535 | Solute carrier family 2, facilitated glucose transporter member 4 | SLC2A4 |
| TAR00011 | Insulin receptor | INSR |
| TAR04107 | CD40 ligand | CD40LG |
| TAR04456 | Prostaglandin E synthase | PTGES |
| TAR04311 | Kinetochore protein Nuf2 | NUF2 |
| TAR04015 | Adenylate cyclase type 2 | ADCY2 |
| TAR00349 | Hepatocyte growth factor receptor | MET |
| TAR03907 | Calmodulin | PCP4 |
| TAR00105 | Alpha-2A adrenergic receptor | ADRA2A |
| TAR00126 | Alpha-2C adrenergic receptor | ADRA2C |
| TAR00037 | Potassium voltage-gated channel subfamily H member 2 | KCNH2 |
| TAR03727 | Calcium-activated potassium channel subunit alpha 1 | KCNMA1 |
| TAR04526 | Signal transducer and activator of transcription 3 | STAT3 |
| TAR04187 | Endothelin-1 | EDN1 |
| TAR00284 | Neuronal acetylcholine receptor subunit alpha-2 | CHRNA2 |
| TAR00499 | Beta-lactamase | LACTBL1 |
| TAR00056 | D(1B) dopamine receptor | DRD5 |
| TAR00123 | Coagulation factor VII | F7 |
| TAR00106 | 5-hydroxytryptamine 1A receptor | HTR1A |
| TAR00141 | Gamma-aminobutyric-acid receptor alpha-2 subunit | GABRA2 |
| TAR00181 | Gamma-aminobutyric-acid receptor alpha-5 subunit | GABRA5 |
| TAR00186 | Sodium-dependent noradrenaline transporter | SLC6A2 |
| TAR00200 | Gamma-aminobutyric-acid receptor alpha-3 subunit | GABRA3 |
| TAR00203 | 5-hydroxytryptamine 2C receptor | HTR2C |
| TAR00214 | Alpha-2B adrenergic receptor | ADRA2B |
| TAR00310 | 5-hydroxytryptamine 1B receptor | HTR1B |
| TAR03284 | Gamma-aminobutyric-acid receptor subunit alpha-6 | GABRA6 |
| TAR03412 | Gamma-aminobutyric acid receptor subunit gamma-3 | GABRG3 |
| TAR03967 | Gamma-aminobutyric acid receptor subunit epsilon | GABRE |
| TAR00086 | Apoptosis regulator Bcl-2 | BCL2 |
| TAR04478 | Proto-oncogene c-Fos | FOS |
| TAR04007 | Activator of 90 kDa heat shock protein ATPase homolog 1 | AHSA1 |
| TAR00374 | Fatty acid synthase | FASN |
| TAR00199 | Endothelin-1 receptor | EDNRA |
| TAR00621 | Cytochrome P450 3A4 | CYP3A4 |
| TAR00724 | Cytochrome P450 1A2 | CYP1A2 |
| TAR04372 | Myc proto-oncogene protein | MYC |
| TAR04149 | Cytochrome P450 1A1 | CYP1A1 |
| TAR04403 | Nuclear receptor subfamily 1 group I member 2 | NR1I2 |
| TAR04406 | Nucleophosmin | NPM1 |
| TAR00706 | Endothelin-converting enzyme 1 | ECE1 |
| TAR04436 | Poly [ADP-ribose] polymerase 4 | PARP4 |
| TAR00168 | Calcitonin receptor | CALCR |
| TAR00459 | Integrin beta-3 | ITGB3 |
| TAR04197 | Eukaryotic translation initiation factor 6 | EIF6 |
| TAR04126 | Collagen alpha-1(VII) chain | COL7A1 |
| TAR00727 | Alcohol dehydrogenase 1C | ADH1C |
| TAR01143 | Cytochrome P450-cam | camA |
| TAR03216 | Glutamate receptor 2 | GRIA2 |
| TAR00726 | Alcohol dehydrogenase 1B | ADH1B |
| TAR01172 | Lysozyme | LYZ |
| TAR02262 | Nicotinate-nucleotide--dimethylbenzimidazole phosphoribosyltransferase | cobT |
| TAR04033 | Apoptosis regulator BAX | BAX |
| TAR04089 | Caspase-8 | CASP8 |
| TAR04469 | Protein kinase C alpha type | PRKCA |
| TAR04568 | Transforming growth factor beta-1 | TGFB1 |
| TAR00357 | Serum paraoxonase/arylesterase 1 | PON1 |
| TAR03987 | Microtubule-associated protein 2 | MAP2 |
| TAR04303 | Interleukin-8 | CXCL8 |
| TAR04470 | Protein kinase C beta type | PRKCB |
| TAR04279 | Insulin-like growth factor II | IGF2 |
| TAR02952 | Glutathione S-transferase Mu 1 | GSTM1 |
| TAR03688 | Glutathione S-transferase Mu 2 | GSTM2 |
| TAR00766 | Glutathione S-transferase A1 | GSTA1 |
| TAR03674 | Glutathione S-transferase A2 | GSTA2 |
| TAR04117 | Chitin synthase 2 | CHS2 |
| TAR04362 | Monocyte differentiation antigen CD14 | CD14 |
| TAR04322 | Lipopolysaccharide-binding protein | LBP |
| TAR04263 | Hypoxia-inducible factor 1-alpha | HIF1A |
| TAR04211 | Fos-related antigen 1 | FOSL1 |
| TAR04212 | Fos-related antigen 2 | FOSL2 |
| TAR00431 | Cell division control protein 2 homolog | CDK1 |
| TAR00428 | Myeloperoxidase | MPO |
| TAR03204 | Aryl hydrocarbon receptor | AHR |
| TAR03611 | Cytochrome c | CYCS |
| TAR04629 | Arachidonate 12-lipoxygenase, 12S-type | ALOX12 |
| TAR04399 | Nuclear factor of activated T-cells, cytoplasmic 1 | NFATC1 |
| TAR04582 | Tudor domain-containing protein 7 | TDRD7 |
| TAR03433 | Egl nine homolog 1 | EGLN1 |
| TAR04381 | NADPH oxidase 5 | NOX5 |
| TAR04200 | Fatty acid-binding protein, epidermal | FABP5 |
| TAR04032 | Apolipoprotein D | APOD |
| TAR00346 | Urokinase-type plasminogen activator | PLAU |
| TAR00521 | Leukotriene A-4 hydrolase | LTA4H |
| TAR00565 | Amine oxidase [flavin-containing] B | MAOB |
| TAR00566 | Amine oxidase [flavin-containing] A | MAOA |
| TAR01696 | Chymotrypsinogen B | CTRB1 |
| TAR00063 | Beta-1 adrenergic receptor | ADRB1 |
| TAR00735 | Catalase | CAT |
| TAR04262 | Hyaluronan synthase 2 | HAS2 |
| TAR03586 | cAMP-dependent protein kinase inhibitor alpha | PKIA |
| TAR00597 | Superoxide dismutase [Cu-Zn] | SOD1 |
| TAR00246 | Cytosolic phospholipase A2 | PLA2G4A |
| TAR04079 | Canalicular multispecific organic anion transporter 1 | ABCC2 |
| TAR00595 | Serine/threonine-protein kinase mTOR | MTOR |
| TAR00441 | Stromelysin-1 | MMP3 |
| TAR00734 | Pro-epidermal growth factor | EGF |
| TAR04144 | Cyclin-dependent kinase inhibitor 2A, isoforms 1/2/3 | CDKN2A |
| TAR04194 | ETS domain-containing protein Elk-1 | ELK1 |
| TAR00744 | NADPH--cytochrome P450 reductase | POR |
| TAR00153 | Ornithine decarboxylase | ODC1 |
| TAR04491 | RAF proto-oncogene serine/threonine-protein kinase | RAF1 |
| TAR04525 | Signal transducer and activator of transcription 1-alpha/beta | STAT1 |
| TAR04464 | Protein CBFA2T1 | RUNX1T1 |
| TAR02963 | 78 kDa glucose-regulated protein | HSPA5 |
| TAR00231 | Acetyl-CoA carboxylase 1 | ACACA |
| TAR04095 | Caveolin-1 | CAV1 |
| TAR00466 | Tissue factor | F3 |
| TAR00436 | Gap junction alpha-1 protein | GJA1 |
| TAR00418 | Interleukin-1 beta | IL1B |
| TAR00417 | C-C motif chemokine 2 | CCL2 |
| TAR00427 | E-selectin | SELE |
| TAR00440 | Vascular cell adhesion protein 1 | VCAM1 |
| TAR00593 | Prostaglandin E2 receptor EP3 subtype | PTGER3 |
| TAR04174 | Dual oxidase 2 | DUOX2 |
| TAR04247 | Heat shock protein beta-1 | HSPB1 |
| TAR00781 | Estrogen sulfotransferase | SULT1E1 |
| TAR00318 | Maltase-glucoamylase, intestinal | MGAM |
| TAR04150 | Cytochrome P450 1B1 | CYP1B1 |
| TAR00434 | Tissue-type plasminogen activator | PLAT |
| TAR00457 | Thrombomodulin | THBD |
| TAR04429 | Plasminogen activator inhibitor 1 | SERPINE1 |
| TAR00731 | Collagen alpha-1(I) chain | COL1A1 |
| TAR00088 | Arachidonate 5-lipoxygenase | ALOX5 |
| TAR04620 | Phosphatidylinositol-3,4,5-trisphosphate 3-phosphatase and dual-specificity protein phosphatase PTEN | PTEN |
| TAR04297 | Interleukin-1 alpha | IL1A |
| TAR04391 | Neutrophil cytosol factor 1 | NCF1 |
| TAR04038 | ATP-binding cassette sub-family G member 2 | ABCG2 |
| TAR04398 | Nuclear factor erythroid 2-related factor 2 | NFE2L2 |
| TAR00470 | NAD(P)H dehydrogenase [quinone] 1 | NQO1 |
| TAR04435 | Poly [ADP-ribose] polymerase 1 | PARP1 |
| TAR03993 | 26S proteasome non-ATPase regulatory subunit 3 | PSMD3 |
| TAR02885 | Collagen alpha-1(III) chain | COL3A1 |
| TAR01366 | DNA gyrase subunit B | gyrB |
| TAR04137 | C-X-C motif chemokine 11 | CXCL11 |
| TAR04138 | C-X-C motif chemokine 2 | CXCL2 |
| TAR04159 | DDB1- and CUL4-associated factor 5 | DCAF5 |
| TAR04404 | Nuclear receptor subfamily 1 group I member 3 | NR1I3 |
| TAR04513 | Serine/threonine-protein kinase Chk2 | CHEK2 |
| TAR04125 | Claudin-4 | CLDN4 |
| TAR04420 | Peroxisome proliferator-activated receptor alpha | PPARA |
| TAR04421 | Peroxisome proliferator-activated receptor delta | PPARD |
| TAR04246 | Heat shock factor protein 1 | HSF1 |
| TAR04130 | C-reactive protein | CRP |
| TAR04136 | C-X-C motif chemokine 10 | CXCL10 |
| TAR04270 | Inhibitor of nuclear factor kappa-B kinase subunit alpha | CHUK |
| TAR04411 | Osteopontin | SPP1 |
| TAR04505 | Runt-related transcription factor 2 | RUNX2 |
| TAR04492 | Ras association domain-containing protein 1 | RASSF1 |
| TAR04560 | Transcription factor E2F1 | E2F1 |
| TAR04561 | Transcription factor E2F2 | E2F2 |
| TAR02998 | Prostatic acid phosphatase | ACPP |
| TAR00363 | Cathepsin D | CTSD |
| TAR04276 | Insulin-like growth factor-binding protein 3 | IGFBP3 |
| TAR04290 | Interferon regulatory factor 1 | IRF1 |
| TAR04497 | Receptor tyrosine-protein kinase erbB-3 | ERBB3 |
| TAR04589 | Type I iodothyronine deiodinase | DIO1 |
| TAR04443 | Procollagen C-endopeptidase enhancer 1 | PCOLCE |
| TAR04484 | Puromycin-sensitive aminopeptidase | NPEPPS |
| TAR04252 | Hexokinase-2 | HK2 |
| TAR04259 | Homeobox protein Nkx-3.1 | NKX3-1 |
| TAR04493 | Ras GTPase-activating protein 1 | RASA1 |
| TAR04418 | Peroxidase C1A | PRXC1A |
| TAR00421 | Mitogen-activated protein kinase 3 | MAPK3 |
| TAR00190 | Low-density lipoprotein receptor | LDLR |
| TAR04050 | Bcl2 antagonist of cell death | BAD |
| TAR04349 | Microsomal triglyceride transfer protein large subunit | MTTP |
| TAR04031 | Apolipoprotein B-100 | APOB |
| TAR04428 | Phospholipase B1, membrane-associated | PLB1 |
| TAR01644 | 3-hydroxy-3-methylglutaryl-coenzyme A reductase | HMGCR |
| TAR04148 | Cytochrome P450 19A1 | CYP19A1 |
| TAR04614 | UDP-glucuronosyltransferase 1-1 | UGT1A8 |
| TAR04543 | Sterol regulatory element-binding protein 1 | SREBF1 |
| TAR00240 | Glutathione reductase, mitochondrial | GSR |
| TAR00306 | Multidrug resistance-associated protein 1 | ABCC1 |
| TAR04017 | Adiponectin | ADIPOQ |
| TAR04542 | Sterol O-acyltransferase 2 | SOAT2 |
| TAR03236 | Aldo-keto reductase family 1 member C1 | AKR1C1 |
| TAR00202 | Aspartate aminotransferase, cytoplasmic | GOT1 |
| TAR00092 | 4-aminobutyrate aminotransferase, mitochondrial | ABAT |
| TAR04325 | Liver carboxylesterase 1 | CES1 |
| TAR03202 | Sterol O-acyltransferase 1 | SOAT1 |
| TAR00139 | Vascular endothelial growth factor receptor 2 | KDR |
| TAR04051 | Bcl-2-binding component 3 | BBC3 |
| TAR04549 | Telomerase protein component 1 | TEP1 |
| TAR04471 | Protein kinase C delta type | PRKCD |
| TAR04206 | Fibronectin | FN1 |
| TAR00477 | Rhodopsin | RHO |
| TAR01306 | Ferrichrome-iron receptor | fhuA |
| TAR00537 | Cell division protein kinase 7 | CDK7 |
| TAR03937 | Cytochrome P450 2C9 | CYP2C9 |
| TAR04475 | Prothrombin | F2 |
