# Supplementary material for: Mechanism of Fei-Xian Formula in the Treatment of Pulmonary Fibrosis on the Basis of Network Pharmacology Analysis Combined with Molecular Docking Validation
Source: Evid Based Complement Alternat Med. 2021 Aug 3;2021:6658395. doi: 10.1155/2021/6658395 (PMC8357467; doi:10.1155/2021/6658395)
Supplement: Supplementary Materials — Table S1: all the pharmacodynamic ingredients of FXF. Table S2: all the potential pharmacodynamic targets of FXF. Table S3: known pulmonary fibrosis-related targets. Table S4: FXF shared 87 potential pharmacodynamic targets with known pulmonary fibrosis-related targets. Table S5: degree values of nodes in the candidate active ingredient-target network of FXF in treating pulmonary fibrosis. [file 6658395.f1.zip › 6658395.f1/Table S3.docx]

| **Table S3. Known pulmonary fibrosis-related targets** | |
| --- | --- |
| **Gene name** | **Annotation** |
| RTEL1 | Regulator Of Telomere Elongation Helicase 1 |
| CFTR | CF Transmembrane Conductance Regulator |
| PARN | Poly(A)-Specific Ribonuclease |
| RTEL1-TNFRSF6B | RTEL1-TNFRSF6B Readthrough (NMD Candidate) |
| BMPR2 | Bone Morphogenetic Protein Receptor Type 2 |
| SFTPB | Surfactant Protein B |
| SFTPA2 | Surfactant Protein A2 |
| SFTPA1 | Surfactant Protein A1 |
| TERC | Telomerase RNA Component |
| CAV1 | Caveolin 1 |
| IL10 | Interleukin 10 |
| TLR4 | Toll Like Receptor 4 |
| CCR6 | C-C Motif Chemokine Receptor 6 |
| MIR21 | MicroRNA 21 |
| MUC1 | Mucin 1, Cell Surface Associated |
| SFTPD | Surfactant Protein D |
| SMAD3 | SMAD Family Member 3 |
| FAM111B | Family With Sequence Similarity 111 Member B |
| TGFBR1 | Transforming Growth Factor Beta Receptor 1 |
| TINF2 | TERF1 Interacting Nuclear Factor 2 |
| TP53 | Tumor Protein P53 |
| AKT1 | AKT Serine/Threonine Kinase 1 |
| STN1 | STN1 Subunit Of CST Complex |
| ENG | Endoglin |
| PRTN3 | Proteinase 3 |
| HLA-DRB1 | Major Histocompatibility Complex, Class II, DR Beta 1 |
| NHP2 | NHP2 Ribonucleoprotein |
| CTNNB1 | Catenin Beta 1 |
| F2 | Coagulation Factor II, Thrombin |
| NOP10 | NOP10 Ribonucleoprotein |
| TGFB2 | Transforming Growth Factor Beta 2 |
| ACVRL1 | Activin A Receptor Like Type 1 |
| ATP11A | ATPase Phospholipid Transporting 11A |
| DPP9 | Dipeptidyl Peptidase 9 |
| DKC1 | Dyskerin Pseudouridine Synthase 1 |
| SERPINH1 | Serpin Family H Member 1 |
| NPHP3 | Nephrocystin 3 |
| FASLG | Fas Ligand |
| ALOX5 | Arachidonate 5-Lipoxygenase |
| KCNK3 | Potassium Two Pore Domain Channel Subfamily K Member 3 |
| ACTC1 | Actin Alpha Cardiac Muscle 1 |
| WRAP53 | WD Repeat Containing Antisense To TP53 |
| SMAD9 | SMAD Family Member 9 |
| RPGRIP1L | RPGRIP1 Like |
| NKX2-1 | NK2 Homeobox 1 |
| FBN1 | Fibrillin 1 |
| TMEM67 | Transmembrane Protein 67 |
| ALB | Albumin |
| CRP | C-Reactive Protein |
| FOXF1 | Forkhead Box F1 |
| MIR130A | MicroRNA 130a |
| MBL2 | Mannose Binding Lectin 2 |
| NPHP1 | Nephrocystin 1 |
| IFNG | Interferon Gamma |
| CC2D2A | Coiled-Coil And C2 Domain Containing 2A |
| MIRLET7D | MicroRNA Let-7d |
| PKHD1 | PKHD1 Ciliary IPT Domain Containing Fibrocystin/Polyductin |
| BMP6 | Bone Morphogenetic Protein 6 |
| NPHP4 | Nephrocystin 4 |
| NEK8 | NIMA Related Kinase 8 |
| REN | Renin |
| SMPD1 | Sphingomyelin Phosphodiesterase 1 |
| CEP290 | Centrosomal Protein 290 |
| GATA4 | GATA Binding Protein 4 |
| TTC21B | Tetratricopeptide Repeat Domain 21B |
| MIR34C | MicroRNA 34c |
| SRC | SRC Proto-Oncogene, Non-Receptor Tyrosine Kinase |
| MKS1 | MKS Transition Zone Complex Subunit 1 |
| JAG1 | Jagged Canonical Notch Ligand 1 |
| WDR19 | WD Repeat Domain 19 |
| HFE | Homeostatic Iron Regulator |
| INVS | Inversin |
| VEGFA | Vascular Endothelial Growth Factor A |
| SERPINC1 | Serpin Family C Member 1 |
| HPS4 | HPS4 Biogenesis Of Lysosomal Organelles Complex 3 Subunit 2 |
| NF1 | Neurofibromin 1 |
| OFD1 | OFD1 Centriole And Centriolar Satellite Protein |
| FCGR2A | Fc Fragment Of IgG Receptor IIa |
| AGTR1 | Angiotensin II Receptor Type 1 |
| SCNN1A | Sodium Channel Epithelial 1 Subunit Alpha |
| TMEM216 | Transmembrane Protein 216 |
| AHI1 | Abelson Helper Integration Site 1 |
| PPARG | Peroxisome Proliferator Activated Receptor Gamma |
| ZCCHC8 | Zinc Finger CCHC-Type Containing 8 |
| TLR2 | Toll Like Receptor 2 |
| NOTCH1 | Notch Receptor 1 |
| TTN | Titin |
| IFT80 | Intraflagellar Transport 80 |
| MPO | Myeloperoxidase |
| CSF2RA | Colony Stimulating Factor 2 Receptor Subunit Alpha |
| TCTN2 | Tectonic Family Member 2 |
| ANKS6 | Ankyrin Repeat And Sterile Alpha Motif Domain Containing 6 |
| B9D1 | B9 Domain Containing 1 |
| CD4 | CD4 Molecule |
| BBS2 | Bardet-Biedl Syndrome 2 |
| KIF7 | Kinesin Family Member 7 |
| NPPB | Natriuretic Peptide B |
| FAS | Fas Cell Surface Death Receptor |
| TNNT2 | Troponin T2, Cardiac Type |
| KRT18 | Keratin 18 |
| BBS1 | Bardet-Biedl Syndrome 1 |
| CXCR3 | C-X-C Motif Chemokine Receptor 3 |
| MYRF | Myelin Regulatory Factor |
| CXCL10 | C-X-C Motif Chemokine Ligand 10 |
| LEP | Leptin |
| GDF1 | Growth Differentiation Factor 1 |
| CSPP1 | Centrosome And Spindle Pole Associated Protein 1 |
| HSPG2 | Heparan Sulfate Proteoglycan 2 |
| HIF1A | Hypoxia Inducible Factor 1 Subunit Alpha |
| KIAA0586 | KIAA0586 |
| NEK9 | NIMA Related Kinase 9 |
| TNFRSF1A | TNF Receptor Superfamily Member 1A |
| HPS3 | HPS3 Biogenesis Of Lysosomal Organelles Complex 2 Subunit 1 |
| HPS5 | HPS5 Biogenesis Of Lysosomal Organelles Complex 2 Subunit 2 |
| THBD | Thrombomodulin |
| TGFB1 | transforming growth factor beta 1 |
| CCN2 | cellular communication network factor 2 |
| NFE2L2 | nuclear factor, erythroid 2 like 2 |
| IL1B | interleukin 1 beta |
| EDN1 | endothelin 1 |
| MMP9 | matrix metallopeptidase 9 |
| CCL2 | C-C motif chemokine ligand 2 |
| IL4 | interleukin 4 |
| IL13 | interleukin 13 |
| TIMP1 | TIMP metallopeptidase inhibitor 1 |
| AGT | angiotensinogen |
| CSF2 | colony stimulating factor 2 |
| HPS1 | HPS1 biogenesis of lysosomal organelles complex 3 subunit 1 |
| FAM13A | family with sequence similarity 13 member A |
| DSP | desmoplakin |
| ABCA3 | ATP binding cassette subfamily A member 3 |
| AP3B1 | adaptor related protein complex 3 subunit beta 1 |
| FN1 | fibronectin 1 |
| TNF | tumor necrosis factor |
| HGF | hepatocyte growth factor |
| IL6 | interleukin 6 |
| HMGB1 | high mobility group box 1 |
| TGFA | transforming growth factor alpha |
| STAT3 | signal transducer and activator of transcription 3 |
| IGF1 | insulin like growth factor 1 |
| FGF2 | fibroblast growth factor 2 |
| CXCL8 | C-X-C motif chemokine ligand 8 |
| MTOR | mechanistic target of rapamycin kinase |
| AREG | amphiregulin |
| SERPINA1 | serpin family A member 1 |
| MMP2 | matrix metallopeptidase 2 |
| SPP1 | secreted phosphoprotein 1 |
| SOD1 | superoxide dismutase 1 |
| CCR2 | C-C motif chemokine receptor 2 |
| CCL11 | C-C motif chemokine ligand 11 |
| FGF1 | fibroblast growth factor 1 |
| STAT6 | signal transducer and activator of transcription 6 |
| MIR326 | microRNA 326 |
| CCL5 | C-C motif chemokine ligand 5 |
| SOD3 | superoxide dismutase 3 |
| EGF | epidermal growth factor |
| BMP7 | bone morphogenetic protein 7 |
| ELN | elastin |
| FGF7 | fibroblast growth factor 7 |
| COL3A1 | collagen type III alpha 1 chain |
| MIR30A | microRNA 30a |
| MIR29C | microRNA 29c |
| CAT | catalase |
| ADIPOQ | adiponectin, C1Q and collagen domain containing |
| CEBPB | CCAAT enhancer binding protein beta |
| MECP2 | methyl-CpG binding protein 2 |
| HMOX1 | heme oxygenase 1 |
| GREM1 | gremlin 1, DAN family BMP antagonist |
| CSF3 | colony stimulating factor 3 |
| CCR3 | C-C motif chemokine receptor 3 |
| SMAD7 | SMAD family member 7 |
| CCL4 | C-C motif chemokine ligand 4 |
| ACTA2 | actin alpha 2, smooth muscle |
| IL5 | interleukin 5 |
| LAMB1 | laminin subunit beta 1 |
| PARP1 | poly(ADP-ribose) polymerase 1 |
| PDGFA | platelet derived growth factor subunit A |
| IL1RN | interleukin 1 receptor antagonist |
| MIR140 | microRNA 140 |
| MIR125A | microRNA 125a |
| SKIL | SKI like proto-oncogene |
| MIR26B | microRNA 26b |
| PTX3 | pentraxin 3 |
| MIR101-1 | microRNA 101-1 |
| CCL3 | C-C motif chemokine ligand 3 |
| MIR10A | microRNA 10a |
| MIR122 | microRNA 122 |
| FYN | FYN proto-oncogene, Src family tyrosine kinase |
| MIR200C | microRNA 200c |
| CMA1 | chymase 1 |
| MIR345 | microRNA 345 |
| MIR378A | microRNA 378a |
| MIR425 | microRNA 425 |
| CALCA | calcitonin related polypeptide alpha |
| CFD | complement factor D |
| SERPINE1 | serpin family E member 1 |
| PDGFB | platelet derived growth factor subunit B |
| CXCL2 | C-X-C motif chemokine ligand 2 |
| IL12B | interleukin 12B |
| CYSLTR2 | cysteinyl leukotriene receptor 2 |
| CEBPA | CCAAT enhancer binding protein alpha |
| EGFR | epidermal growth factor receptor |
| IL17A | interleukin 17A |
| PTGS2 | prostaglandin-endoperoxide synthase 2 |
| AGER | advanced glycosylation end-product specific receptor |
| COL1A1 | collagen type I alpha 1 chain |
| PLAU | plasminogen activator, urokinase |
| ACE | angiotensin I converting enzyme |
| SMAD4 | SMAD family member 4 |
| IL1A | interleukin 1 alpha |
| CD36 | CD36 molecule |
| ELANE | elastase, neutrophil expressed |
| TGFBR2 | transforming growth factor beta receptor 2 |
| COL1A2 | collagen type I alpha 2 chain |
| SCGB1A1 | secretoglobin family 1A member 1 |
| MMP1 | matrix metallopeptidase 1 |
| MFN2 | mitofusin 2 |
| STAT1 | signal transducer and activator of transcription 1 |
| CDH1 | cadherin 1 |
| PDGFRA | platelet derived growth factor receptor alpha |
| CCL22 | C-C motif chemokine ligand 22 |
| CCL17 | C-C motif chemokine ligand 17 |
| ICAM1 | intercellular adhesion molecule 1 |
| HSP90AB1 | heat shock protein 90 alpha family class B member 1 |
| ANXA1 | annexin A1 |
| HABP2 | hyaluronan binding protein 2 |
| NKX2-5 | NK2 homeobox 5 |
| BAD | BCL2 associated agonist of cell death |
| MUC5AC | mucin 5AC, oligomeric mucus/gel-forming |
| SFTPC | surfactant protein C |
| HSPD1 | heat shock protein family D (Hsp60) member 1 |
| B3GAT1 | beta-1,3-glucuronyltransferase 1 |
| CCR8 | C-C motif chemokine receptor 8 |
| CYSLTR1 | cysteinyl leukotriene receptor 1 |
| VWF | von Willebrand factor |
| MUC5B | mucin 5B, oligomeric mucus/gel-forming |
| CCR4 | C-C motif chemokine receptor 4 |
| TERT | telomerase reverse transcriptase |
| BAX | Apoptosis regulator BAX |
| RELA | Transcription factor p65 |
| BCL2 | Apoptosis regulator Bcl-2 |
| CASP3 | Caspase-3 |
| NOS2 | Nitric oxide synthase, inducible |
| CDKN1A | Cyclin-dependent kinase inhibitor 1 |
| MAPK1 | Mitogen-activated protein kinase 1 |
| NFKBIA | NF-kappa-B inhibitor alpha |
| CXCL1 | Growth-regulated alpha protein |
| MAPK3 | Mitogen-activated protein kinase 3 |
| SOD2 | Superoxide dismutase [Mn], mitochondrial |
| JUN | Transcription factor AP-1 |
| CASP9 | Caspase-9 |
| PCNA | Proliferating cell nuclear antigen |
| DDIT3 | DNA damage-inducible transcript 3 protein |
| GSR | Glutathione reductase, mitochondrial |
| HSPA5 | 78 kDa glucose-regulated protein |
| FOS | Proto-oncogene c-Fos |
| NFKB1 | Nuclear factor NF-kappa-B p105 subunit |
| VCAM1 | Vascular cell adhesion protein 1 |
| CASP8 | Caspase-8 |
| GSK3B | Glycogen synthase kinase-3 beta |
| CCND1 | G1/S-specific cyclin-D1 |
| CD14 | Monocyte differentiation antigen CD14 |
| MAPK8 | Mitogen-activated protein kinase 8 |
| CYP1A1 | Cytochrome P450 1A1 |
| EGR1 | Early growth response protein 1 |
| BIRC5 | Baculoviral IAP repeat containing 5 |
| SQSTM1 | Sequestosome-1 |
| VIM | Vimentin |
| MYC | Myc proto-oncogene protein |
| GPT | Alanine aminotransferase 1 |
| ATF3 | Cyclic AMP-dependent transcription factor ATF-3 |
| F3 | Tissue factor |
| CASP1 | Caspase-1 |
| GADD45A | Growth arrest and DNA damage-inducible protein GADD45 alpha |
| MAPK9 | Mitogen-activated protein kinase 9 |
| TNFSF10 | Tumor necrosis factor ligand superfamily member 10 |
| SELE | E-selectin |
| CXCL5 | C-X-C motif chemokine 5 |
| NQO1 | NAD(P)H dehydrogenase [quinone] 1 |
| NOX4 | NADPH oxidase 4 |
| CYCS | Cytochrome c |
| GPX1 | Glutathione peroxidase 1 |
| CXCL3 | C-X-C motif chemokine 3 |
| GCLC | Glutamate-cysteine ligase catalytic subunit |
| CXCR4 | C-X-C chemokine receptor type 4 |
| IL18 | Interleukin-18 |
| CCL20 | C-C motif chemokine 20 |
| MAP1LC3B | Microtubule-associated proteins 1A/1B light chain 3B |
| FOSL1 | Fos-related antigen 1 |
| IRS1 | Insulin receptor substrate 1 |
| BIRC3 | Baculoviral IAP repeat-containing protein 3 |
| BCL2L1 | Bcl-2-like protein 1 |
| CCNA2 | Cyclin-A2 |
| H2AX | Histone H2AX |
| TXNRD1 | Thioredoxin reductase 1, cytoplasmic |
| IL2 | Interleukin-2 |
| CDK4 | Cyclin-dependent kinase 4 |
| BMP2 | Bone morphogenetic protein 2 |
| CDK2 | Cyclin-dependent kinase 2 |
| PPARA | Peroxisome proliferator-activated receptor alpha |
| THBS1 | Thrombospondin-1 |
| CD44 | CD44 antigen |
| CDKN1B | Cyclin-dependent kinase inhibitor 1B |
| HBEGF | Proheparin-binding EGF-like growth factor |
| CCNB1 | G2/mitotic-specific cyclin-B1 |
| LCN2 | Neutrophil gelatinase-associated lipocalin |
| CXCL12 | Stromal cell-derived factor 1 |
| DUSP1 | Dual specificity protein phosphatase 1 |
| HSPA1A | Heat shock 70 kDa protein 1A |
| GSTP1 | Glutathione S-transferase P |
| ARG1 | Arginase-1 |
| MAPK14 | Mitogen-activated protein kinase 14 |
| FASN | Fatty acid synthase |
| BCL2L11 | Bcl-2-like protein 11 |
| BBC3 | Bcl-2-binding component 3 |
| CCND2 | G1/S-specific cyclin-D2 |
| CASP7 | Caspase-7 |
| G6PD | Glucose-6-phosphate 1-dehydrogenase |
