# Supplementary material for: Mechanism of Fei-Xian Formula in the Treatment of Pulmonary Fibrosis on the Basis of Network Pharmacology Analysis Combined with Molecular Docking Validation
Source: Evid Based Complement Alternat Med. 2021 Aug 3;2021:6658395. doi: 10.1155/2021/6658395 (PMC8357467; doi:10.1155/2021/6658395)
Supplement: Supplementary Materials — Table S1: all the pharmacodynamic ingredients of FXF. Table S2: all the potential pharmacodynamic targets of FXF. Table S3: known pulmonary fibrosis-related targets. Table S4: FXF shared 87 potential pharmacodynamic targets with known pulmonary fibrosis-related targets. Table S5: degree values of nodes in the candidate active ingredient-target network of FXF in treating pulmonary fibrosis. [file 6658395.f1.zip › 6658395.f1/Table S4.docx]

| **Table 4. FXF shared 87 potential pharmacodynamic targets with known pulmonary fibrosis-related targets** | | |
| --- | --- | --- |
| **String ID** | **Gene Names** | **Annoation** |
| 9606.ENSP00000389814 | ADIPOQ | Adiponectin |
| 9606.ENSP00000451828 | AKT1 | RAC-alpha serine/threonine-protein kinase |
| 9606.ENSP00000363512 | ALOX5 | Arachidonate 5-lipoxygenase |
| 9606.ENSP00000378040 | BAD | Bcl2-associated agonist of cell death |
| 9606.ENSP00000293288 | BAX | Apoptosis regulator BAX |
| 9606.ENSP00000404503 | BBC3 | Bcl-2-binding component 3 |
| 9606.ENSP00000381185 | BCL2 | Apoptosis regulator Bcl-2 |
| 9606.ENSP00000302564 | BCL2L1 | Bcl-2-like protein 1 |
| 9606.ENSP00000301633 | BIRC5 | Baculoviral IAP repeat containing 5 |
| 9606.ENSP00000311032 | CASP3 | Caspase-3 |
| 9606.ENSP00000358327 | CASP7 | Caspase-7 |
| 9606.ENSP00000351273 | CASP8 | Caspase-8 |
| 9606.ENSP00000330237 | CASP9 | Caspase-9 |
| 9606.ENSP00000241052 | CAT | Catalase |
| 9606.ENSP00000339191 | CAV1 | Caveolin-1 |
| 9606.ENSP00000225831 | CCL2 | C-C motif chemokine 2 |
| 9606.ENSP00000274026 | CCNA2 | Cyclin-A2 |
| 9606.ENSP00000256442 | CCNB1 | G2/mitotic-specific cyclin-B1 |
| 9606.ENSP00000227507 | CCND1 | G1/S-specific cyclin-D1 |
| 9606.ENSP00000304236 | CD14 | Monocyte differentiation antigen CD14 |
| 9606.ENSP00000266970 | CDK2 | Cyclin-dependent kinase 2 |
| 9606.ENSP00000257904 | CDK4 | Cyclin-dependent kinase 4 |
| 9606.ENSP00000384849 | CDKN1A | Cyclin-dependent kinase inhibitor 1 |
| 9606.ENSP00000225964 | COL1A1 | Collagen alpha-1(I) chain |
| 9606.ENSP00000304408 | COL3A1 | Collagen alpha-1(III) chain |
| 9606.ENSP00000255030 | CRP | C-reactive protein |
| 9606.ENSP00000305651 | CXCL10 | C-X-C motif chemokine 10 |
| 9606.ENSP00000427279 | CXCL2 | C-X-C motif chemokine 2 |
| 9606.ENSP00000306512 | CXCL8 | Interleukin-8 |
| 9606.ENSP00000307786 | CYCS | Cytochrome c |
| 9606.ENSP00000369050 | CYP1A1 | Cytochrome P450 1A1 |
| 9606.ENSP00000368683 | EDN1 | Endothelin-1 |
| 9606.ENSP00000265171 | EGF | Pro-epidermal growth factor |
| 9606.ENSP00000275493 | EGFR | Epidermal growth factor receptor |
| 9606.ENSP00000308541 | F2 | Prothrombin |
| 9606.ENSP00000334145 | F3 | Tissue factor |
| 9606.ENSP00000304592 | FASN | Fatty acid synthase |
| 9606.ENSP00000346839 | FN1 | Fibronectin type III domain containing |
| 9606.ENSP00000306245 | FOS | Proto-oncogene c-Fos |
| 9606.ENSP00000310170 | FOSL1 | Fos-related antigen 1 |
| 9606.ENSP00000324806 | GSK3B | Glycogen synthase kinase-3 beta |
| 9606.ENSP00000221130 | GSR | Glutathione reductase, mitochondrial |
| 9606.ENSP00000381607 | GSTP1 | Glutathione S-transferase P |
| 9606.ENSP00000437955 | HIF1A | Hypoxia-inducible factor 1-alpha |
| 9606.ENSP00000216117 | HMOX1 | Heme oxygenase 1 |
| 9606.ENSP00000324173 | HSPA5 | 78 kDa glucose-regulated protein |
| 9606.ENSP00000264832 | ICAM1 | Intercellular adhesion molecule 1 |
| 9606.ENSP00000229135 | IFNG | Interferon gamma |
| 9606.ENSP00000412237 | IL10 | Interleukin-10 |
| 9606.ENSP00000263339 | IL1A | Interleukin-1 alpha |
| 9606.ENSP00000263341 | IL1B | Interleukin-1 beta |
| 9606.ENSP00000226730 | IL2 | Interleukin-2 |
| 9606.ENSP00000231449 | IL4 | Interleukin-4 |
| 9606.ENSP00000385675 | IL6 | Interleukin-6 |
| 9606.ENSP00000360266 | JUN | Transcription factor AP-1 |
| 9606.ENSP00000215832 | MAPK1 | Mitogen-activated protein kinase 1 |
| 9606.ENSP00000229795 | MAPK14 | Mitogen-activated protein kinase 14 |
| 9606.ENSP00000263025 | MAPK3 | Mitogen-activated protein kinase 3 |
| 9606.ENSP00000322788 | MMP1 | Interstitial collagenase |
| 9606.ENSP00000219070 | MMP2 | 72 kDa type IV collagenase |
| 9606.ENSP00000361405 | MMP9 | Matrix metalloproteinase-9 |
| 9606.ENSP00000225275 | MPO | Myeloperoxidase |
| 9606.ENSP00000354558 | MTOR | Serine/threonine-protein kinase mTOR |
| 9606.ENSP00000479618 | MYC | Myc proto-oncogene protein |
| 9606.ENSP00000380252 | NFE2L2 | Nuclear factor erythroid 2-related factor 2 |
| 9606.ENSP00000216797 | NFKBIA | NF-kappa-B inhibitor alpha |
| 9606.ENSP00000327251 | NOS2 | Nitric oxide synthase, inducible |
| 9606.ENSP00000319788 | NQO1 | NAD(P)H dehydrogenase [quinone] 1 |
| 9606.ENSP00000355759 | PARP1 | Poly [ADP-ribose] polymerase 1 |
| 9606.ENSP00000368458 | PCNA | Proliferating cell nuclear antigen |
| 9606.ENSP00000361850 | PLAU | Urokinase-type plasminogen activator |
| 9606.ENSP00000385523 | PPARA | Peroxisome proliferator-activated receptor alpha |
| 9606.ENSP00000287820 | PPARG | Peroxisome proliferator-activated receptor gamma |
| 9606.ENSP00000356438 | PTGS2 | Prostaglandin G/H synthase 2 |
| 9606.ENSP00000384273 | RELA | Transcription factor p65 |
| 9606.ENSP00000331736 | SELE | E-selectin |
| 9606.ENSP00000223095 | SERPINE1 | Plasminogen activator inhibitor 1 |
| 9606.ENSP00000270142 | SOD1 | Superoxide dismutase [Cu-Zn] |
| 9606.ENSP00000378517 | SPP1 | Osteopontin |
| 9606.ENSP00000354394 | STAT1 | Signal transducer and activator of transcription 1-alpha/beta |
| 9606.ENSP00000264657 | STAT3 | Signal transducer and activator of transcription 3 |
| 9606.ENSP00000221930 | TGFB1 | Transforming growth factor beta-1 |
| 9606.ENSP00000366307 | THBD | Thrombomodulin |
| 9606.ENSP00000398698 | TNF | Tumor necrosis factor |
| 9606.ENSP00000269305 | TP53 | Cellular tumor antigen p53 |
| 9606.ENSP00000294728 | VCAM1 | Vascular cell adhesion protein 1 |
| 9606.ENSP00000478570 | VEGFA | Vascular endothelial growth factor A |
