# Supplementary material for: Mechanism of Fei-Xian Formula in the Treatment of Pulmonary Fibrosis on the Basis of Network Pharmacology Analysis Combined with Molecular Docking Validation
Source: Evid Based Complement Alternat Med. 2021 Aug 3;2021:6658395. doi: 10.1155/2021/6658395 (PMC8357467; doi:10.1155/2021/6658395)
Supplement: Supplementary Materials — Table S1: all the pharmacodynamic ingredients of FXF. Table S2: all the potential pharmacodynamic targets of FXF. Table S3: known pulmonary fibrosis-related targets. Table S4: FXF shared 87 potential pharmacodynamic targets with known pulmonary fibrosis-related targets. Table S5: degree values of nodes in the candidate active ingredient-target network of FXF in treating pulmonary fibrosis. [file 6658395.f1.zip › 6658395.f1/Table S5.docx]

| **Table S5. Degree values of nodes in the candidate active ingredient-target network of FXF in treating pulmonary fibrosis** | |
| --- | --- |
| **Name** | **Degree** |
| MOL000098 | 136 |
| PTGS2 | 88 |
| NOS2 | 77 |
| MOL000358 | 70 |
| CDK2 | 69 |
| GSK3B | 68 |
| CCNA2 | 61 |
| PPARG | 55 |
| MAPK14 | 47 |
| MOL002714 | 42 |
| MOL000006 | 36 |
| MOL001002 | 27 |
| MOL000173 | 24 |
| MOL004328 | 20 |
| MOL007154 | 19 |
| CASP3 | 15 |
| MOL000449 | 15 |
| BCL2 | 14 |
| RELA | 13 |
| MOL007088 | 12 |
| MOL000546 | 11 |
| MOL002928 | 11 |
| BAX | 10 |
| CDKN1A | 10 |
| JUN | 10 |
| AKT1 | 9 |
| CASP9 | 9 |
| MMP9 | 9 |
| VEGFA | 9 |
| TP53 | 8 |
| MOL000492 | 8 |
| CASP8 | 7 |
| GSTP1 | 7 |
| NFKBIA | 7 |
| TGFB1 | 7 |
| MOL000493 | 7 |
| MOL001320 | 7 |
| MOL001368 | 7 |
| MOL001601 | 7 |
| MOL001735 | 7 |
| MOL002651 | 7 |
| MOL003044 | 7 |
| MOL003896 | 7 |
| MOL006992 | 7 |
| MOL007041 | 7 |
| MOL007049 | 7 |
| MOL007058 | 7 |
| MOL007059 | 7 |
| MOL007061 | 7 |
| MOL007068 | 7 |
| MOL007100 | 7 |
| MOL007101 | 7 |
| MOL007127 | 7 |
| MOL007130 | 7 |
| MOL007156 | 7 |
| MOL012101 | 7 |
| MOL012108 | 7 |
| MOL013058 | 7 |
| CCNB1 | 6 |
| CCND1 | 6 |
| CXCL8 | 6 |
| IL6 | 6 |
| MMP2 | 6 |
| TNF | 6 |
| MOL002776 | 6 |
| MOL007036 | 6 |
| MOL007045 | 6 |
| MOL007048 | 6 |
| MOL007050 | 6 |
| MOL007069 | 6 |
| MOL007071 | 6 |
| MOL007079 | 6 |
| MOL007082 | 6 |
| MOL007093 | 6 |
| MOL007094 | 6 |
| MOL007155 | 6 |
| FOS | 5 |
| HIF1A | 5 |
| PLAU | 5 |
| MOL004004 | 5 |
| MOL007105 | 5 |
| MOL007108 | 5 |
| MOL007111 | 5 |
| MOL007119 | 5 |
| MOL007124 | 5 |
| MOL007125 | 5 |
| MOL007136 | 5 |
| MOL007150 | 5 |
| MOL007151 | 5 |
| MOL007152 | 5 |
| BCL2L1 | 4 |
| BIRC5 | 4 |
| MAPK1 | 4 |
| MPO | 4 |
| SOD1 | 4 |
| MOL001352 | 4 |
| MOL007098 | 4 |
| MOL013059 | 4 |
| CAT | 3 |
| CCL2 | 3 |
| CYP1A1 | 3 |
| EGFR | 3 |
| FASN | 3 |
| HMOX1 | 3 |
| ICAM1 | 3 |
| IFNG | 3 |
| IL10 | 3 |
| IL2 | 3 |
| MMP1 | 3 |
| MYC | 3 |
| PPARA | 3 |
| MOL000569 | 3 |
| MOL001924 | 3 |
| MOL007120 | 3 |
| MOL007122 | 3 |
| MOL007143 | 3 |
| MOL007145 | 3 |
| ALOX5 | 2 |
| CAV1 | 2 |
| COL1A1 | 2 |
| COL3A1 | 2 |
| CRP | 2 |
| CXCL10 | 2 |
| CXCL2 | 2 |
| CYCS | 2 |
| EDN1 | 2 |
| EGF | 2 |
| F3 | 2 |
| FOSL1 | 2 |
| HSPA5 | 2 |
| IL1A | 2 |
| IL1B | 2 |
| NFE2L2 | 2 |
| NQO1 | 2 |
| PARP1 | 2 |
| SELE | 2 |
| SERPINE1 | 2 |
| SPP1 | 2 |
| STAT1 | 2 |
| THBD | 2 |
| VCAM1 | 2 |
| MOL000296 | 2 |
| MOL001328 | 2 |
| MOL001340 | 2 |
| MOL001358 | 2 |
| MOL001494 | 2 |
| MOL001942 | 2 |
| MOL002222 | 2 |
| MOL007064 | 2 |
| MOL007081 | 2 |
| MOL007141 | 2 |
| MOL009754 | 2 |
| BAD | 1 |
| BBC3 | 1 |
| CASP7 | 1 |
| CD14 | 1 |
| CDK4 | 1 |
| F2 | 1 |
| FN1 | 1 |
| GSR | 1 |
| IL4 | 1 |
| MAPK3 | 1 |
| MTOR | 1 |
| PCNA | 1 |
| ADIPOQ | 1 |
| STAT3 | 1 |
| MOL001323 | 1 |
| MOL001329 | 1 |
| MOL001353 | 1 |
| MOL001355 | 1 |
| MOL001361 | 1 |
| MOL002883 | 1 |
| MOL007077 | 1 |
| MOL007085 | 1 |
| MOLHirudin | 1 |
